# Modulation of mTOR Within Retinal Pigment Epithelium Affects Cell Viability and Mitochondrial Pathology

**DOI:** 10.3390/ijms26199442

**Published:** 2025-09-26

**Authors:** Gloria Lazzeri, Michela Ferrucci, Paola Lenzi, Maria Anita Giambelluca, Francesca Biagioni, Carla Letizia Busceti, Alessandro Frati, Francesco Fornai

**Affiliations:** 1 Human Anatomy, Department of Translational Research and New Technologies in Medicine and Surgery, University of Pisa, 56126 Pisa, Italy; gloria.lazzeri@unipi.it (G.L.); michela.ferrucci@unipi.it (M.F.); paola.lenzi@unipi.it (P.L.); maria.giambelluca@unipi.it (M.A.G.); 2 IRCCS Neuromed, 86077 Pozzilli, Italy; francesca.biagioni@neuromed.it (F.B.); carla.busceti@neuromed.it (C.L.B.); alessandro.frati@neuromed.it (A.F.); 3 Neurosurgery Division, Human Neurosciences Department, Sapienza University, 00135 Rome, Italy

**Keywords:** mitochondrial morphology, mitochondrial ultrastructure, autophagy, curcumin, rapamycin, PINK1, Parkin, Tomm20, MitoTracker Green, MitoTracker Red, ZO-1

## Abstract

The relevance of well-structured mitochondria in sustaining the integrity of the retinal pigment epithelium (RPE) is increasingly evident. Conversely, altered mitochondria are a culprit of age-related macular degeneration (AMD), which is influenced by the activity of mechanistic target of rapamycin (mTOR). In the present manuscript, the mitochondrial status of RPE cells was investigated by light and electron microscopy following the administration of various doses of compounds, which modulate mTOR. The study combines MitoTracker dyes and mitochondrial immunohistochemistry with in situ mitochondrial morphometry. Various doses of 3-methyladenine (3-MA), curcumin, and rapamycin were administered alone or in combination. The activity of autophagy and mTOR was quantified following each treatment. Administration of 3-MA led to activation of mTOR, which was associated with severe cell death, altered membrane permeability, and altered ZO-1 expression. In this condition, mitochondrial mass was reduced, despite a dramatic increase in damaged mitochondria being reported. The decrease in healthy mitochondria was concomitant with alterations in key mitochondria-related antigens such as Tomm20, Pink1, and Parkin. Specific mitochondrial alterations were quantified through in situ ultrastructural morphometry. Both curcumin and rapamycin counteract mTOR activation and rescue mitochondrial status, while preventing RPE cell loss and misplacement of decreased ZO-1 expression. Mitigation of mTOR may protect mitochondria in retinal degeneration.

## 1. Introduction

Increasing evidence indicates that, in the retina, activation of the mechanistic target of rapamycin (mTOR) may produce a number of effects, including dysfunctional autophagy, which are critical to promote retinal degeneration as witnessed by a number of retinal disorders [1,2,3,4,5]. These include both acute injuries and chronic diseases, where a number of alterations are reported both at the experimental level [6,7,8,9,10,11] and in human persons [12,13,14]. Although this concept applies to a vast number of disorders, most of the evidence is obtained from chronic retinal degenerative diseases. Among these, the most common is represented by age-related macular degeneration (AMD) [12,13,15,16,17]. This is relevant for the general population since AMD represents the prevalent cause of blindness among degenerative retinal diseases. The cell types, which are mostly involved in the course of AMD, correspond to those layered at the border between the outer retina and inner choroid. Thus, AMD early affects photoreceptors, retinal pigment epithelium (RPE), Bruch’s membrane, and vessels within the inner choroid, which altogether represent the retinal neurovascular unit (RNVU) [13,17,18,19,20,21,22,23,24,25]. Although baseline mTOR activity is critical in all these cells, there is no doubt that the cell type that is mostly affected corresponds to retinal pigment epithelium (RPE). This is because RPE is in constant need of a powerful clearing system to remove a number of abnormal chemical species and altered organelles, which are produced during photoreception. This is also connected with the massive energy requirement of RPE cells, where multiple mitochondria are challenged to neutralize reactive oxygen species (ROS) and sustain a high turnover of lipids, sugars, and proteins [26,27,28,29,30,31]. Within RPE, a well-represented glucose-sensing activity addresses sugars towards aerobic metabolism [32,33,34,35,36,37,38,39,40,41], and a powerful enzymatic activity is involved in lipid metabolism [42,43,44,45]. In keeping with this, a main homeostatic function of RPE cells is manifest by glycophagy and lipophagy. This occurs through the enzymatic digestion of glycogen granules and lipid droplets [15,46]. It is reported that, while in healthy conditions, RPE metabolism fully relies on mitochondrial oxidative metabolism, in AMD, anaerobic energy production becomes noticeable. This finding is consistent with the observation that mitochondrial activity in AMD is severely impaired [47]. Similarly, the loss of energy production from sugars and lipids leads to the accumulation of glycogen granules and lipid droplets along with altered mitochondria within AMD RPE [48]. This scenario is consistent with a defective mitochondrial status as a key determinant in producing AMD, as discussed extensively in a very recent publication [44].

Mitochondrial dynamics are markedly affected by mTOR, and more specifically by mTOR complex 1 (mTORC1), which is allosterically inhibited by rapamycin [49,50,51,52,53,54,55]. Many effects produced by mTORC1 are bound to autophagy modulation [56,57,58,59]. mTOR complex 2 (mTORC2) is less sensitive to rapamycin [60] and its effects are less powerful on autophagy modulation, including mitophagy and mitochondrial turnover, being involved in slightly different activities [61,62,63]. Nonetheless, recent studies indicate that mTORC2 also acts as an autophagy inhibitor [53,64,65,66,67]. In particular, mTORC2 inhibits mitophagy, thus resulting in mitochondrial alterations [68,69].

The most powerful effect is due to mTORC1. In fact, activation of mTORC1 suppresses mitophagy and increases the amount of altered mitochondria, which accumulate within stagnant autophagy vacuoles [70,71]. This worsens mitochondrial function and morphology [70,71,72,73,74,75,76]. In contrast, mTORC1 activates mitochondrial fission while inhibiting mitochondrial fusion, which contributes to producing mitochondrial alterations [70,77,78,79,80,81,82].

Therefore, it is expected that mTORC1 (hereafter referred to as mTOR) activation produces mitochondrial alterations along with a loss of viability of RPE cells. In the present study, we analyzed whether mTOR activation may produce RPE cell damage and mitochondrial alterations mimicking those occurring during AMD. These are assessed both at light and electron microscopy. The study is carried out using Adult Retinal Pigment Epithelial cell line-19 (ARPE-19), where activation of mTOR is produced by different doses of 3-methyladenine (3-MA), while mTOR inhibition was produced by rapamycin [83,84], or the phytochemical curcumin.

Briefly, 3-MA acts primarily as an inhibitor of the enzyme class III phosphatidyl-inositol-3-kinase (PI3K) [85,86,87]. This leads to autophagy suppression and mTOR activation [88,89]. Following 3-MA, cytotoxicity is associated with defective removal of damaged cell components, including dysfunctional mitochondria [13,90,91].

Curcumin is a bioactive polyphenolic compound derived from the rhizomes of Curcuma longa (turmeric), a plant of the ginger family (*Zingiberaceae*) [92,93]. Curcumin extracts exhibit beneficial effects in a wide range of pathological conditions, including inflammation, skin disorders, cancer, as well as cardiovascular and neurological diseases [93,94,95,96,97,98,99,100].

Curcumin exerts its protective effects by acting as an antioxidant and anti-inflammatory agent and autophagy promoter [101,102,103,104]. In detail, via downregulation of the PI3K/Akt/mTOR pathway, curcumin stimulates mitophagy and preserves mitochondrial morphology [102,105,106,107,108].

In detail, cytopathology was measured directly by using the classic Hematoxylin &Eosin (H&E) staining, or indirectly through dyes depending on the integrity of the cell membrane. Immunohistochemistry and electron microscopy, including the stoichiometry of specific proteins, were carried out. The modulation of mTOR activity was assessed by measuring the amount of downstream enzyme product P70 S6 Kinase (PS6K) both by densitometry and quantitative stoichiometry. The integrity of RPE was further assessed by staining the ZO-1 protein, which marks the contour of healthy RPE. The involvement of mitochondria was investigated through histofluorescent dyes, MitoTrackers, Green and Red (MTR-G and MTR-red, respectively), along with Tomm20, Pink1, and Parkin immunohistochemistry carried out alone or in combination, and further validated by the count of mitochondria ultrastructural morphometry.

## 2. Results

### 2.1. In RPE Cells, 3-MA Suppresses, While Curcumin and Rapamycin Promote Cell Viability

**Figure 1:**
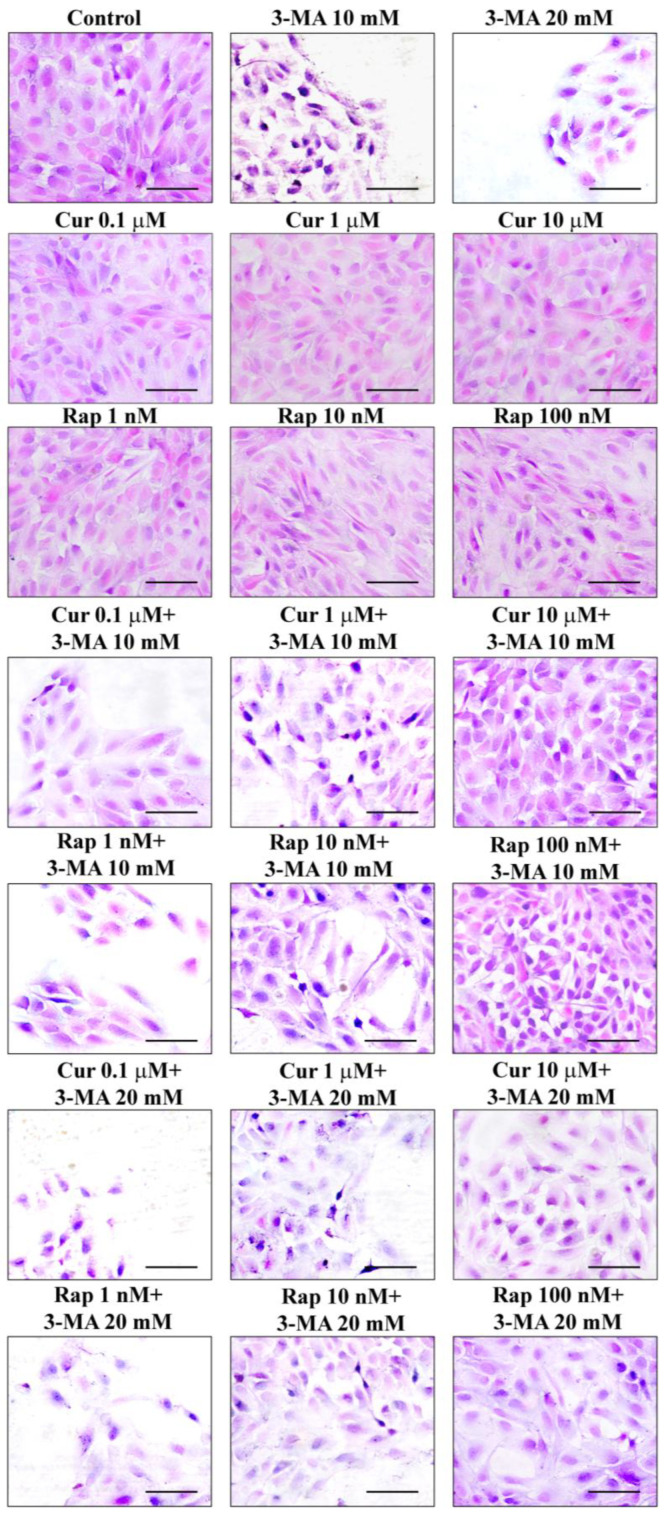
Curcumin and rapamycin dose-dependently prevent RPE cell degeneration induced by 3-MA. Representative pictures of H&E-stained RPE cells in baseline conditions (controls) and following treatments with increasing doses of curcumin (0.1 μM, 1 μM, 10 μM) or rapamycin (1 nM, 10 nM, 100 nM), administered alone or in combination with 3-MA (10 mM or 20 mM). Control RPE cells are represented by a quite continuous mono-layer, where cells adhere to each other. Cells own a prevalent polygonal shape, with a moderately eosin-stained cytosol and hematoxylin-stained nucleus. Administration of the autophagy inhibitor 3-MA produces dose-dependent cell loss, which is accompanied by severe alterations, mainly consisting of abnormal cell size and pale vacuolar cytosolic areas. Curcumin or rapamycin, when administered alone, do not produce any significant effect on morphology or H&E staining of RPE cells. Co-administration of curcumin or rapamycin with 3-MA protects against 3-MA-induced cell damage. Scale bars = 66 μm.

**Figure 2:**
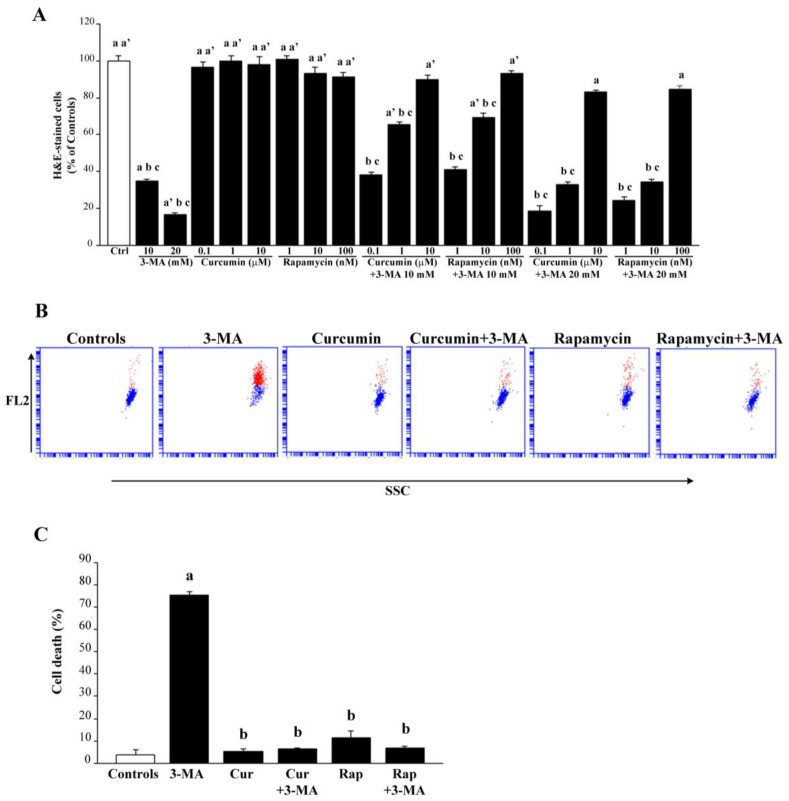
Curcumin and rapamycin dose-dependently prevent RPE cell loss induced by 3-MA. Graph in (**A**) reports the percentage of H&E-stained RPE cells following treatments with increasing doses of curcumin (0.1 mM, 1 mM, 10 mM) or rapamycin (1 nM, 10 nM, 100 nM), administered alone or in combination with 3-MA (10 mM or 20 mM). (**B**) Representative cytometry plot graphs show the effects on cell death, which was evaluated by using incorporation of propidium iodide. Cells were cells treated with curcumin (10 μM) or rapamycin (100 nM), administered alone or in combination with 3-MA (20 mM). Blue plots represent viable cells; red plots represent dead cells. Graph (**C**) reports the percentage of dead cells. Values are expressed as: (**A**) the mean percentage ± S.E.M. (assuming controls = 100%), (**C**) the mean percentage ± S.E.M. out of total cells, from three independent experiments. Ctrl = controls; Cur = curcumin; FL2 = Fluorescence channel 2; Rap = rapamycin; SSC = side scatter channel. ^a^ *p* < 0.05 compared with 3-MA 20 mM; ^a’^ *p* < 0.05 compared with 3-MA 10 mM; ^b^ *p* < 0.05 compared with single treatments; ^c^ *p* < 0.05 compared with controls.

According to representative pictures in Figure 1, H&E staining of control cells provided quite homogeneous cell areas and shapes with a prevalent polygonal feature, with some cell processes shortly extending from polygon angles. These control cells grow to form a continuous mono-layer and adhere through their cell contours. When 3-MA is administered at the doses of 10 mM and 20 mM, a progressive loss of cell number was observed (representative Figure 1 and graph of Figure 2A). Apart from producing cell loss, in the cell culture (Figure 1) 3-MA increases the number of frankly degenerating cells (up to 10%). The severe cell damage assessed by H&E is based on the actual lack of cell structures and the presence of severe alterations. This concerns abnormal cell size and shape, cytosol staining, and nuclear staining, frankly differing from standard control cells, even considering those undergoing spontaneous degeneration within control cultures.

**Figure 3:**
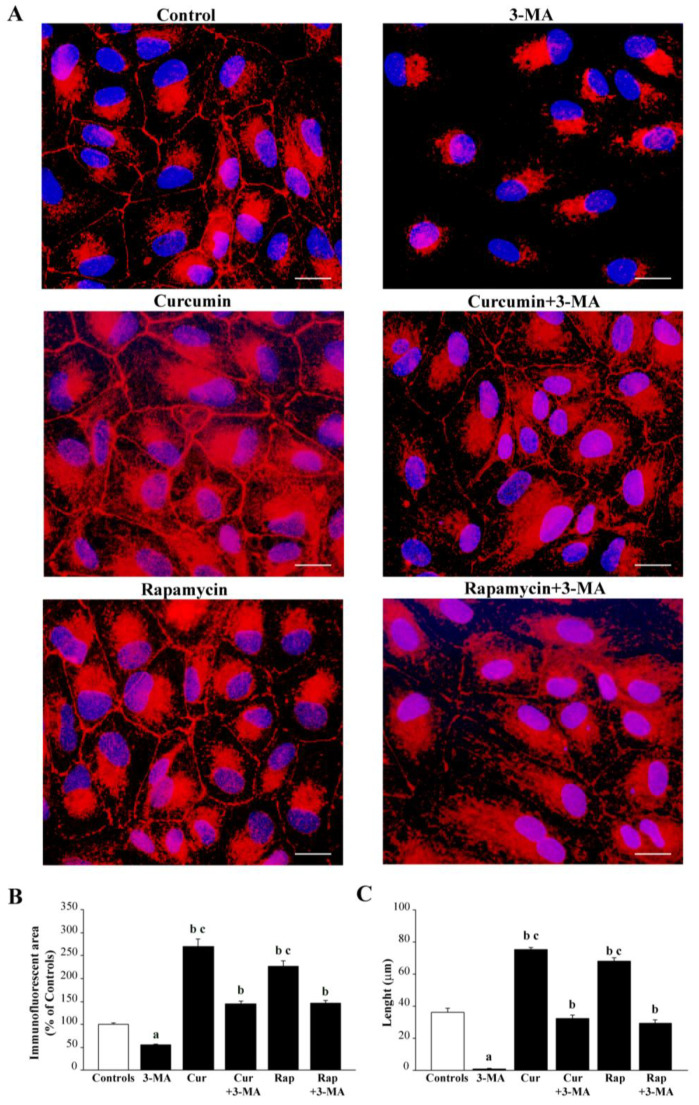
Curcumin and rapamycin counteract the dismantling of ZO-1 immunofluorescence induced by 3-MA. (**A**) Representative pictures of ZO-1 immunofluorescence in control RPE cells and cells following treatment with 3-MA (10 mM), curcumin (10 μM), rapamycin (100 nM), and a combination of curcumin+3-MA or rapamycin+3-MA. Administration of 3-MA reduces ZO-1 immunofluorescence mostly along the cell contour. In contrast, curcumin or rapamycin, when administered alone, increase ZO-1 immunofluorescence, both in the whole cell and mainly along the cell contour. When co-administered with 3-MA, both curcumin and rapamycin prevent the loss of ZO-1 immunopositive cell area and cell contour induced by 3-MA. The graphs report (**B**) the ZO-1 immunofluorescent area and (**C**) the ZO-1 immunopositive cell contour, measured in each experimental group. Values are expressed as the mean percentage ± S.E.M. of the ZO-1 immunofluorescent area (assuming controls = 100%) or the mean ± S.E.M. of the ZO-1 immunopositive cell contour (in μm) measured in N = 60 cells per experimental group from three independent experiments. Cur = curcumin; Rap = rapamycin. ^a^ *p* < 0.05 compared with all other groups; ^b^ *p* < 0.05 compared with 3-MA; ^c^ *p* < 0.05 compared with controls. Scale bars = 17 μm.

Degenerating cells feature pale vacuolar cytosolic areas, irregular shape, and smaller size compared with the homogenously stained cytosol and regular cell shape of the control. When counting cell viability in the graph of Figure 2A, the percentage of viable H&E-stained cells was reported for all groups, assuming controls = 100%. The damage produced by 3-MA and the rescue achieved by co-administration of various doses of curcumin or rapamycin were reported in the graph of Figure 2A. This shows that 3-MA at the dose of 10 mM produces less than 70% of cell loss, while the dose of 20 mM produces an excess of 80% of cell loss. However, the dose of 10 mM produces a significant amount of degenerating cells (as described above and shown in representative pictures of Figure 1), while the dose of 3-MA, 20 mM, produced a higher amount of cell loss and a decreased amount of borderline, degenerating cells. The effects of various compounds on cell viability were replicated by assessing the pathological membrane permeability. In detail, the cell staining following exposure with the DNA-binding compound propidium iodide, replicates data obtained by counting cell viability with classic H&E staining (graph in Figure 2A,B). The staining with propidium iodide is less direct than H&E to assess cell pathology; it rather provides information concerning the disruption of membrane impermeability, which often anticipates cell death. When cell pathology was inferred by the loss of immunostaining for the functional protein ZO-1, 3-MA administered at the dose of 10 mM produced a loss of cell contour, which surpassed 95% of controls and which was in excess compared with the loss of ZO-1 immunofluorescent cell area (roughly 45%, representative pictures of Figure 3A and graph of Figure 3B). Administration of curcumin or rapamycin, when given alone, increases roughly two-fold ZO-1 immunostaining counted in control cells, both concerning the cell contour and the cell area (Figure 3A–C). Rapamycin alone, at the dose of 100 nM, produced effects that were similar to those induced by curcumin concerning ZO-1 immunopositive cell contour and cell area. Again, curcumin and rapamycin were both effective in preventing the loss of ZO-1 immunostaining induced by 3-MA compared with controls (Figure 3A–C). These protective effects were complete since no statistical difference between the combined treatment and controls could be noticed.

**Figure 4:**
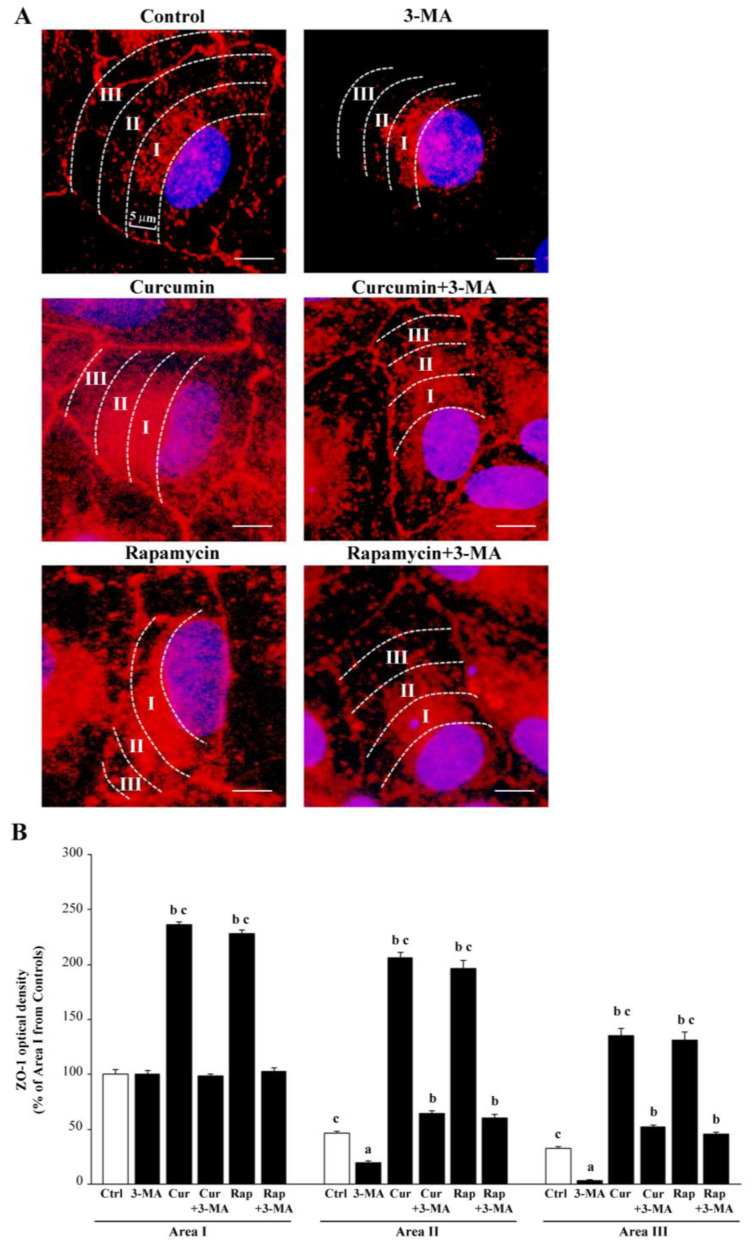
Representative intracellular gradient of ZO-1 immunofluorescence. (**A**) Representative pictures of ZO-1 immunofluorescence from a control cell and cells treated with 3-MA (10 mM), curcumin (10 μM), rapamycin (100 nM), and a combination of curcumin+3-MA or rapamycin+3-MA. Dotted white lines were drawn at 5 μm from the nucleus (Area I) or 5 μm from each other (Area II and Area III) to select three distinct cytosolic regions, which were indicated as peri-nuclear (Area I), intermediate (Area II), and peri-membranous (Area III) cytosolic areas. Following 3-MA, despite a drastic loss of ZO-1, as reported in Figure 3, there is a marked discrepancy depending on cytosolic domains. Therefore, within peri-nuclear Area I, ZO-1 immunofluorescence is comparable with control, while a dramatic drop is observed within intermediate Area II and mostly within peri-membranous Area III. This effect is prevented when curcumin or rapamycin is co-administered with 3-MA. Remarkably, this gradient of immunostaining is representative of distinct cell domains, where deleterious or protective effects take place. In detail, 3-MA, curcumin, or rapamycin do not exert an effect, since ZO-1 immunofluorescence is distributed homogeneously in the cell. Therefore, rather than measuring the ZO-1 protein in the whole cell, we expressed the alteration of the ZO-1 intracellular pattern following each treatment. The graph in (**B**) reports the extent of the ZO-1 immunofluorescence measured within each selected area. Values are reported as the mean percentage ± S.E.M. of the immunofluorescent areas (assuming Area I within controls = 100%) from N = 60 cells per group from three independent experiments. For each experimental group, the cell shown here was selected from representative pictures shown in Figure 3. Ctrl = controls; Cur = curcumin; Rap = rapamycin. ^a^ *p* < 0.05 compared with all other groups; ^b^ *p* < 0.05 compared with 3-MA; ^c^ *p* < 0.05 compared with controls. Scale bars = 7 μm.

Treatment with 3-MA, curcumin, or rapamycin also modified the zonal pattern of fluorescence per cell. This implies that, following 3-MA, red immunofluorescence of ZO-1 was clustered and densely packed in a perinuclear position, whereas in control cells, immunofluorescence was spread according to a pattern where a radial gradient from the nucleus to the cell membrane could be measured (representative Figure 4A and graph of Figure 4B). As evidenced in Figure 3 and further detailed in representative Figure 4, 3-MA actually reduces the ZO-1 immunostaining in the whole cell; however, the suppression of peri-membranous placement of ZO-1 is mostly evident. When counting immunodensity within arbitrarily selected cell areas, the peri-nuclear Area I was at least as fluorescent in 3-MA-treated cells as in controls. Area II was fluorescent in controls, while it was empty following 3-MA. Similarly, the peri-membranous Area III, which includes the cell contour, was still fluorescent in controls, while it was invisible following 3-MA.

The administration of either curcumin or rapamycin increased immunofluorescence in each Area. When curcumin or rapamycin were co-administered with 3-MA, the loss of immunofluorescence was significantly counteracted, mostly concerning Area I and Area III.

Conversely, when rapamycin or curcumin is administered, either alone or in combination with 3-MA, the clusterization and packaging of the proteins in the perinuclear position is counteracted, and the placement of the protein around the nucleus and periphery (up to the cell contour) is re-established. This mimics a critical process occurring in the course of AMD, where the dismantling of proteins placed on the cell membrane to form intercellular junctions is more and more evident in the disease course. In fact, in AMD, the loss of physiological activity of RPE cells is suddenly evident by the dismantling of those proteins such as ZO-1, claudin, and occludin, which are critical to sustain the planar arrangement of RPE cells, impeding the entry of molecules within the retina. Again, such a dismantling is correlated with the shift in AMD from a dry into a wet isoform, fostered by the epithelial–mesenchymal transition (EMT) of RPE cells, which may produce neo-angiogenesis. In the present study, we demonstrated that the effects of 3-MA, which dismantles ZO-1 from RPE cells, are counteracted by curcumin and rapamycin, which reassemble ZO-1 on the cell membrane.

### 2.2. In RPE Cells 3-MA Suppresses, While Curcumin or Rapamycin Promotes Mitochondrial Integrity

#### 2.2.1. Evidence from Histochemistry (MTR-G and MTR-R)

**Figure 5:**
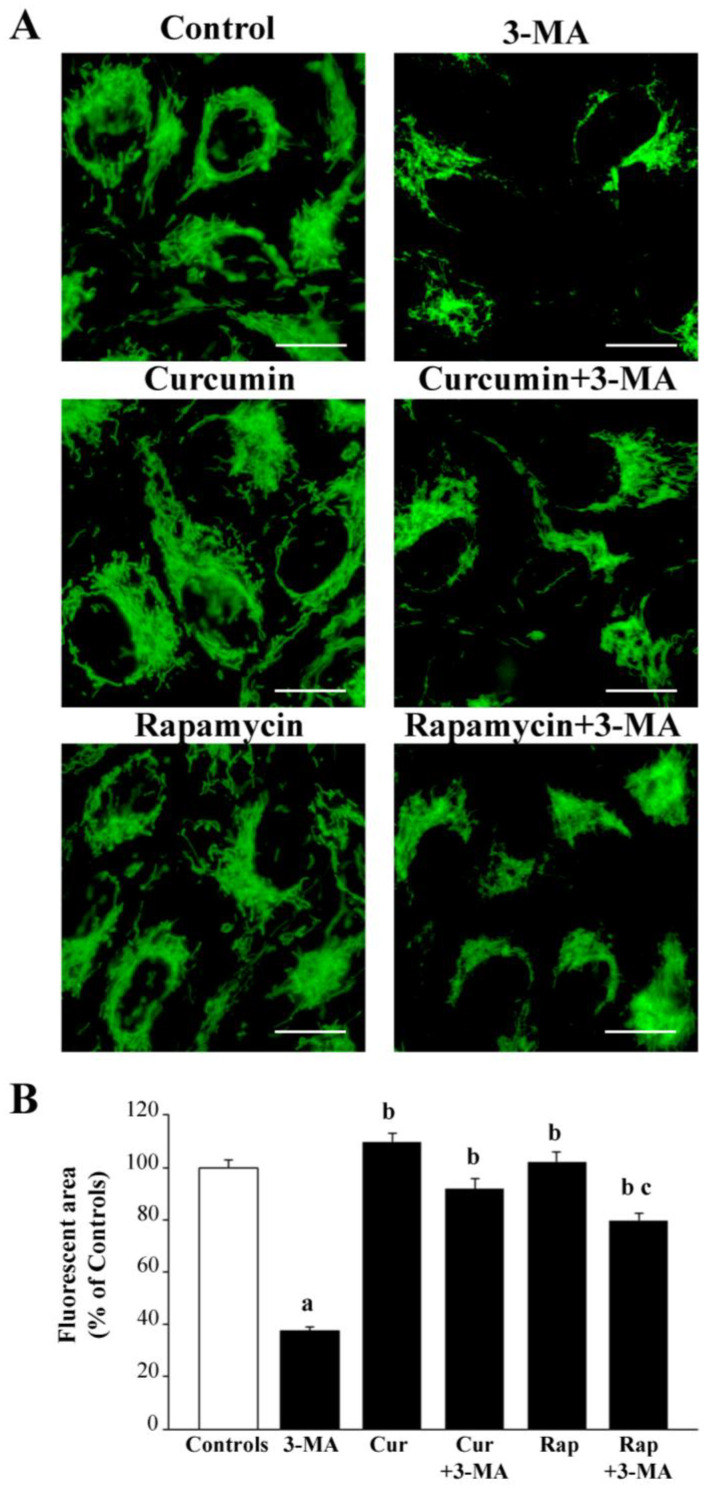
Curcumin and rapamycin prevent 3-MA-induced decrease in MTR-G fluorescence. (**A**) Representative pictures of MTR-G fluorescent cells in baseline conditions and following treatment with 3-MA (10 mM), curcumin (10 μM), or rapamycin (100 nM) alone or in combination. Administration of 3-MA suppresses MTR-G fluorescence, which stains the mitochondrial mass of RPE cells. Curcumin or rapamycin, when administered alone, does not produce any noticeable effect compared with controls, while the combined administration of either curcumin or rapamycin with 3-MA counteracts the decrease induced by 3-MA. The graph in (**B**) reports the MTR-G fluorescent area measured in each experimental group. Values are expressed as the mean percentage ± S.E.M. of the MTR-G fluorescent area (assuming controls = 100%) measured in N = 60 cells per experimental group, from three independent experiments. Cur = curcumin; Rap = rapamycin. ^a^ *p* < 0.05 compared with all other groups; ^b^ *p* < 0.05 compared with 3-MA; ^c^ *p* < 0.05 compared with controls. Scale bars = 20 μm.

Mitochondrial imaging carried out at light microscopy with the aid of the mitochondrial dyes MTR-G and MTR-R produces quite different results. In fact, as mentioned, MTR-G stains all mitochondria independently of their integrity and occurrence of mitochondrial alterations [109,110,111,112], while MTR-R is a dye that stains healthy mitochondria owing to a normal architecture [113,114,115,116]. Results obtained following MTR-G indicate that the amount of the whole mitochondrial mass per cell is dramatically reduced following 3-MA, since histofluorescence (expressed by the mean fluorescent area) was reduced to less than 50% of controls (representative pictures of Figure 5A and graph of Figure 5B). This is reminiscent of the decrease in the number of mitochondria, which is described within RPE cells in the course of AMD [44]. The effects of either curcumin or rapamycin administered alone were not noticeable compared with controls, while the combined administration of either curcumin or rapamycin with 3MA counteracted the decrease in mitochondrial mass (representative pictures of Figure 5A and graph of Figure 5B).

**Figure 6:**
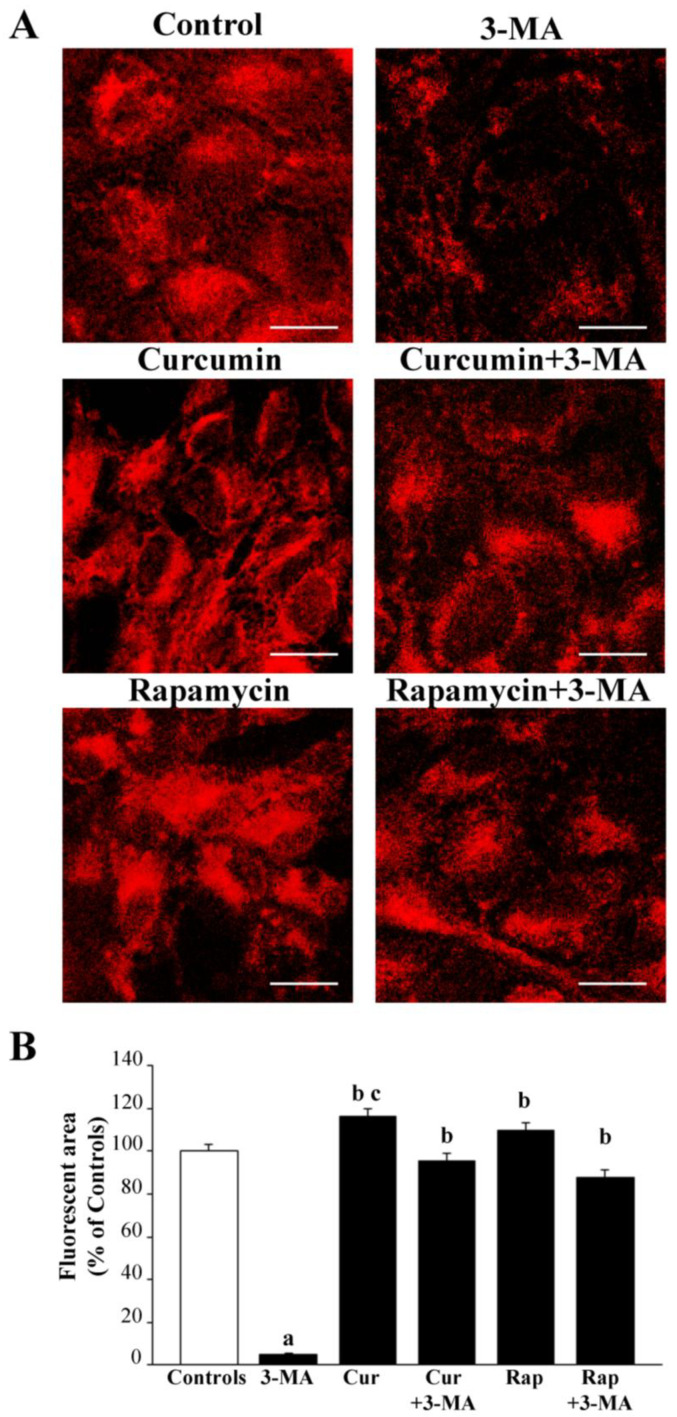
Curcumin and rapamycin prevent 3-MA-induced decrease in MTR-R fluorescence. (**A**) Representative pictures of MTR-R fluorescent cells in baseline conditions and following treatment with 3-MA (10 mM), curcumin (10 μM), or rapamycin (100 nM), alone or in combination. MTR-R fluorescence, which labels healthy mitochondria, is dramatically reduced following 3-MA administration. Curcumin or rapamycin rescues the integrity of the mitochondrial mass. The graph in (**B**) reports the MTR-R fluorescent area measured in each experimental group. Values are expressed as the mean percentage ± S.E.M. of the MTR-R fluorescent area (assuming controls = 100%) measured in N = 60 cells per experimental group from three independent experiments. Cur = curcumin; Rap = rapamycin. ^a^ *p* < 0.05 compared with all other groups; ^b^ *p* < 0.05 compared with 3-MA; ^c^ *p* < 0.05 compared with controls. Scale bars = 20 μm.

These findings suggest that, within RPE cells, 3-MA decreases mitochondrial biogenesis, which is more relevant than the suppression of mitochondrial removal. In fact, when the MTR-R dye was used to stain healthy mitochondria (representative pictures of Figure 6A, and graph of Figure 6B), the effects of 3-MA indicate a drastic suppression of integrity in the mitochondrial mass.

Again, both curcumin and rapamycin, when administered in combination with 3-MA, counteract these effects, bringing back the mass of healthy mitochondria to values similar to controls. This suggests a net effect of both curcumin and rapamycin to stimulate the biogenesis of mitochondria, since the removal of altered mitochondria would not affect mitochondrial mass stained with MTR-R. In fact, when administered alone, both curcumin and rapamycin increased the mass of healthy mitochondria above the values measured in control cells (curcumin being more effective than rapamycin). This point represents a peculiarity of RPE cells, since most cell lines and neuronal phenotypes undergo a net increase in the whole mitochondrial mass following 3-MA administration. In the case of RPE cells, the mitochondrial removal is peculiar, being less dependent on canonical mTOR-dependent autophagy compared with mitochondrial biogenesis [44].

#### 2.2.2. Evidence from Immunofluorescence (Tomm20, Pink1, Parkin Immunostaining)

Immunofluorescence for the constitutive mitochondrial protein Tomm20 was consistent with data produced by the histofluorescence for MTR-G. This also applies to data measuring the actual fluorescent cell area, and when the fluorescent cell area was expressed as a percentage of controls. In detail, 3-MA reduces immunofluorescence for Tomm20 to less than 50% of controls, just like it was measured for MTR-G. This corresponds to a mean fluorescent area of 90.3 ± 3.2 μm^2^ measured in cells administered 3-MA compared with 300.5 ± 7.6 μm^2^ measured in control cells (as reported in Supplementary Table S1). When the rough area is calculated for MTR-G, the corresponding values are the following: mean fluorescent area of 119.3 ± 4.9 μm^2^ measured in cells administered 3-MA compared with 316.1 ± 11.0 μm^2^ measured in control cells (as reported in Supplementary Table S1). This correspondence validates the significance of Tomm20 as an index of whole mitochondria mass independently of mitochondrial integrity. A gold-standard validation is expected by the paragraph reporting data from ultrastructural morphometry of mitochondria.

**Figure 7:**
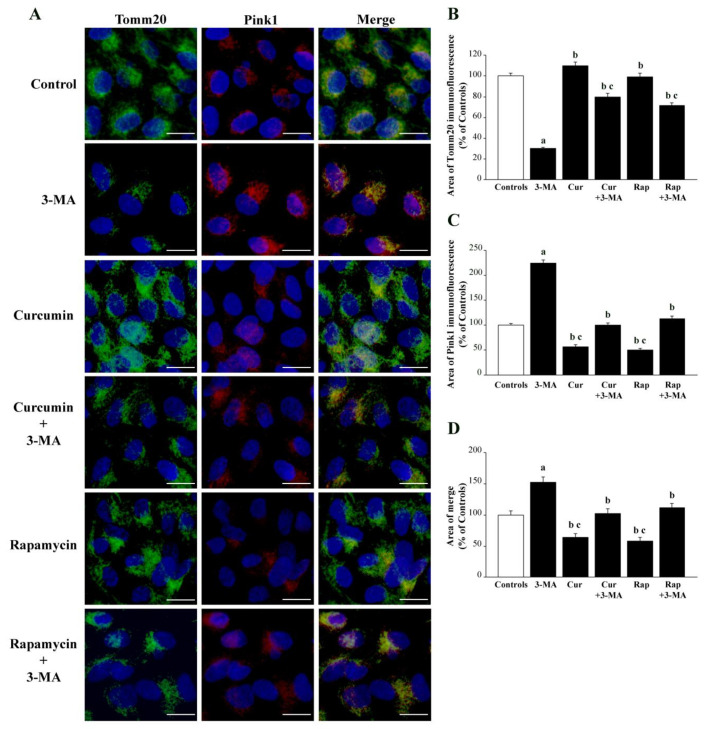
Effects of 3-MA, curcumin, or rapamycin on Tomm20 and Pink1 immunofluorescence. (**A**) Representative pictures of immunofluorescence for Tomm20, Pink1, and their merging in baseline conditions and following treatment with 3-MA (10 mM), curcumin (10 μM), rapamycin (100 nM), alone or in combination. Administration of 3-MA reduces Tomm20 immunofluorescence compared with controls. Neither curcumin nor rapamycin, when administered alone, alters Tomm20 immunofluorescence compared with controls, while co-administration of curcumin or rapamycin with 3-MA counteracts the effect produced by 3-MA. Conversely, Pink1 immunofluorescence is increased after 3-MA treatment, and it is reduced following administration of curcumin or rapamycin alone, whereas combined treatment with curcumin+3-MA or rapamycin+3-MA counteracts the increase in Pink1 immunofluorescence induced by 3-MA. When Tomm20 immunofluorescence is merged with Pink1 immunofluorescence, in 3-MA-treated cells, the merging area is increased compared with controls, and clear and intense merging spots are evident within the cytosol. Curcumin or rapamycin decreases Tomm20 and Pink1 merging, while combined administration of curcumin or rapamycin with 3-MA counteracts the effect of 3-MA. The graphs report the measure of (**B**) Tomm20 immunofluorescent area, (**C**) Pink1 immunofluorescent area, and (**D**) Tomm20+Pink1 merging area. Each area was expressed as mean percentage ± S.E.M. measured in N = 60 cells per experimental group from three independent experiments, assuming controls = 100%. Cur = curcumin; Rap = rapamycin. ^a^ *p* < 0.05 compared with all other groups; ^b^ *p* < 0.05 compared with 3-MA; ^c^ *p* < 0.05 compared with controls. Scale bars = 25 μm.

The combination of Tomm20 with Pink1. When Pink1 immunostaining was carried out (Figure 7A), a marked increase in cell fluorescence was detected following 3-MA. This is in line with the original findings of Lenzi et al. [117] obtained by silencing or overexpressing Pink1 in the course of mTOR alterations. Mitochondrial alterations leading to mitochondrial removal are strongly dependent on the expression at the mitochondrial level of the protein Pink1, which attracts parkin to promote mitochondrial removal via autophagy and proteasome [117,118,119,120,121,122,123,124,125]. The expression of Pink1 is visible in the representative pictures of Figure 7A, and it is counted in the graph of Figure 7C. The expression of Pink1, which was increased by 3-MA, was reduced below the levels of controls when either curcumin or rapamycin was administered alone. In cells receiving the combined administration of either curcumin or rapamycin with 3-MA, the marked increase in Pink1 immunofluorescence was occluded (Figure 7A and graph of Figure 7C). When the expression of Pink1 was assessed in combination with Tomm20, the administration of 3-MA produced a clear and intense merging area, as expected by combining a mitochondrial staining (Tomm20) with a staining specific for damaged mitochondria (Pink1). No other treatment was able to produce such an evident merging. Indeed, when curcumin or rapamycin was administered, the increase in the mitochondria mass was less dependent on altered mitochondria, which generated a less definite merging area (Representative Figure 7A and graph of Figure 7D).

**Figure 8:**
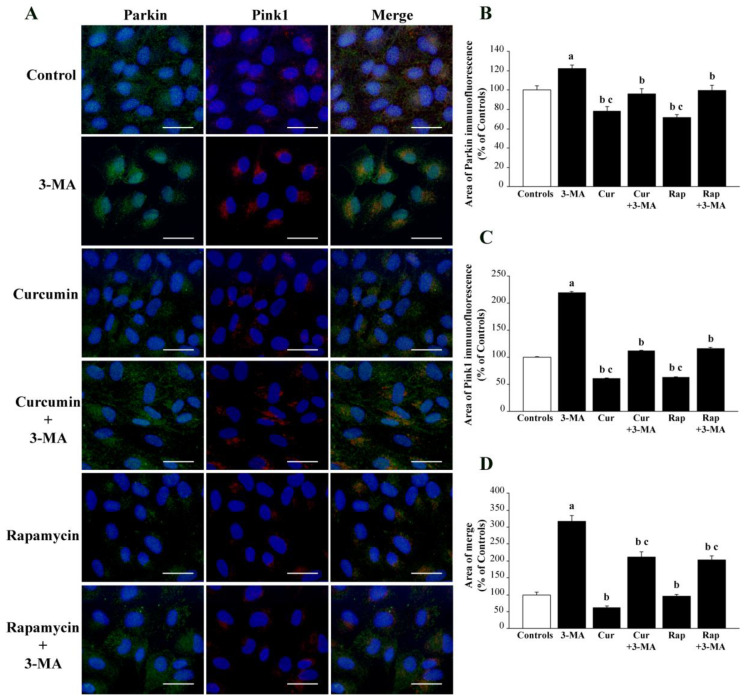
Effects of 3-MA, curcumin, or rapamycin on Parkin and Pink1 immunofluorescence. (**A**) Representative pictures of immunofluorescence for Parkin, Pink1, and their merging in baseline conditions and following treatment with 3-MA (10 mM), curcumin (10 μM), rapamycin (100 nM), alone or in combination. Parkin immunofluorescence increases following administration of 3-MA, while it is reduced following curcumin or rapamycin administration. Both curcumin and rapamycin suppress the effects of 3-MA. The merging between Parkin and Pink1 immunofluorescence is greatly increased following 3-MA. Both curcumin and rapamycin suppress this effect. The graphs report the measure of (**B**) Parkin immunofluorescent area, (**C**) Pink1 immunofluorescent area, and (**D**) Parkin+Pink1 merging area. Each area was measured in N = 60 cells per experimental group from three independent experiments, and it was expressed as mean percentage ± S.E.M., assuming controls = 100%. Cur = curcumin; Rap = rapamycin. ^a^ *p* < 0.05 compared with all other groups; ^b^ *p* < 0.05 compared with 3-MA; ^c^ *p* < 0.05 compared with controls. Scale bars = 38 μm.

The combination of Parkin with Pink1. When Parkin immunostaining was carried out, administration of 3-MA increased immunofluorescence, which was otherwise suppressed by concomitant exposure to either curcumin or rapamycin (representative pictures of Figure 8A and graph of Figure 8B). This is expected considering the bulk of mTOR substrates, which vary following mTOR modulation, requiring Parkin as a protein to promote cell clearance. The merging between Pink1 and Parkin specifically stains the mitochondrial mass, where Pink1, acting as a sensor for mitochondrial alterations, interacts with Parkin to promote mitochondrial removal. Following 3-MA, the merging massively increased to an extent that was not suppressed by either curcumin or rapamycin administration (representative pictures of Figure 8A and graph of Figure 8D). This suggests that mitochondrial removal within RPE cells may depend on a pathway alternative to Pink1-Parkin interaction, such as Beclin1 and AMBRA1 [44,126,127].

In any case, inference from immunofluorescence concerning mitochondrial status following various treatments of RPE cells remains uncertain due to the limits of morphological resolution inherent to immunofluorescence. Thus, this is further addressed by in situ ultrastructural morphometry at TEM.

#### 2.2.3. Evidence from TEM and Ultrastructural Morphometry

**Figure 9:**
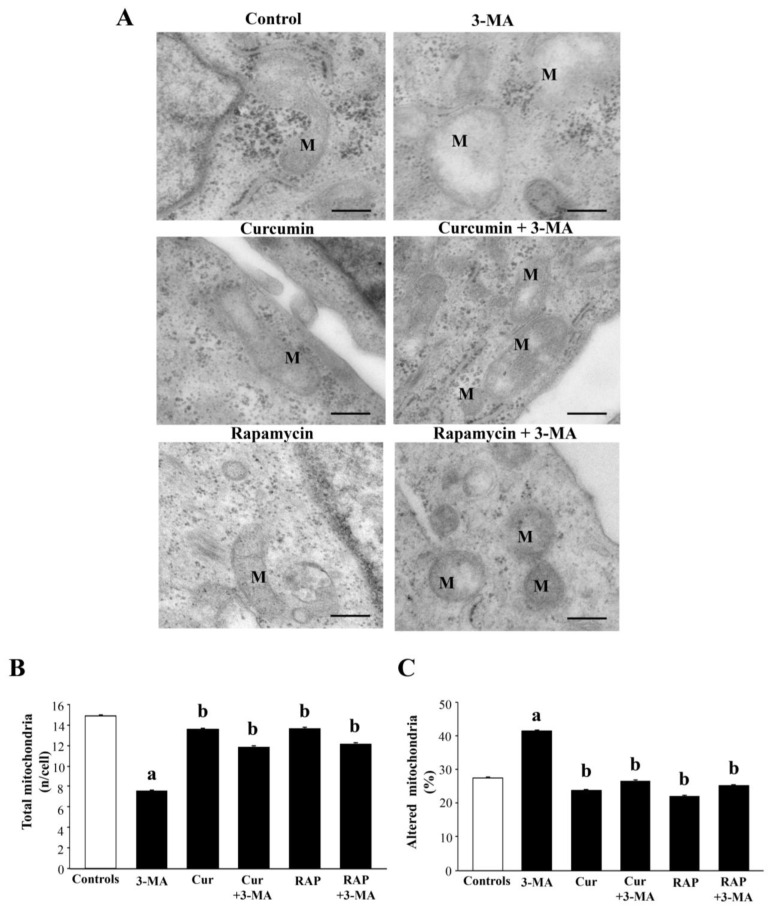
Ultrastructural mitochondrial morphometry within RPE cells. (**A**) Representative micrographs showing the abundance and the status of mitochondria from controls, 3-MA (10 mM), curcumin (10 μM), or rapamycin (100 nM), alone or in combination. Apart from decreasing mitochondria, 3-MA generates several mitochondrial alterations, which match the criteria for the definition of mitochondrial damage. This effect is counteracted by curcumin or rapamycin. The graphs report (**B**) the number of total mitochondria, and (**C**) the percentage of altered mitochondria, measured in N = 40 cells per experimental group. Values are expressed as the mean ± S.E.M. of the number of total mitochondria and as mean percentage ± S.E.M. of altered mitochondria (assuming controls = 100%). Cur = curcumin; M = mitochondria; Rap = rapamycin. ^a^ *p* < 0.05 compared with all other groups; ^b^ *p* < 0.05 compared with 3-MA. Scale bars = 380 nm.

As shown in representative pictures of Figure 9A, the amount of total mitochondria was decreased following mTOR activation, while mTOR inhibition per se did not produce any significant effect. When either curcumin or rapamycin was combined with 3-MA, the decrease in total mitochondria was occluded (graph in Figure 9B). The count of altered mitochondria was reported as the percentage of total mitochondria in the graph of Figure 9C. Administration of 3-MA significantly increases the percentage of altered mitochondria, which is brought back to control values following co-administration of either curcumin or rapamycin (graph of Figure 9C).

**Figure 10:**
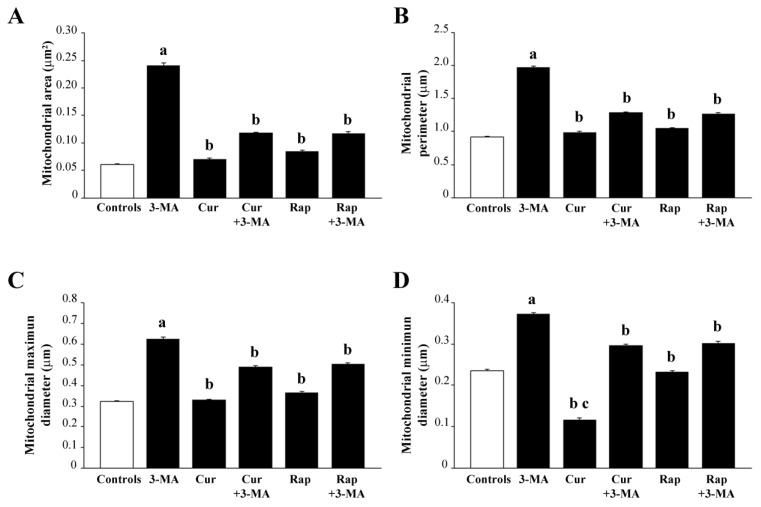
Modulation of mitochondrial size and shape (ultrastructural morphometry). The graphs report: (**A**) mitochondrial area; (**B**) mitochondrial perimeter; (**C**) maximum mitochondrial diameter; (**D**) minimum mitochondrial diameter. Values are expressed as the mean ± S.E.M. measured in N = 30 cells per group. Cur = curcumin; Rap = rapamycin. ^a^ *p* < 0.05 compared with all other groups; ^b^ *p* < 0.05 compared with 3-MA; ^c^ *p* < 0.05 compared with controls.

In detail, as appearing in representative Figure 9A, and reported in the graphs of Figure 10, 3-MA increases mitochondrial area (Figure 10A) and perimeter (Figure 10B), which were consistent with the increase in both maximum and minimum mitochondrial diameter (graphs in Figure 10C and Figure 10D, respectively). Such an effect on mitochondrial size and shape is prevented by co-administration of either curcumin or rapamycin. When administered alone, curcumin reduced the minimum mitochondrial diameter, even compared with controls.

**Figure 11:**
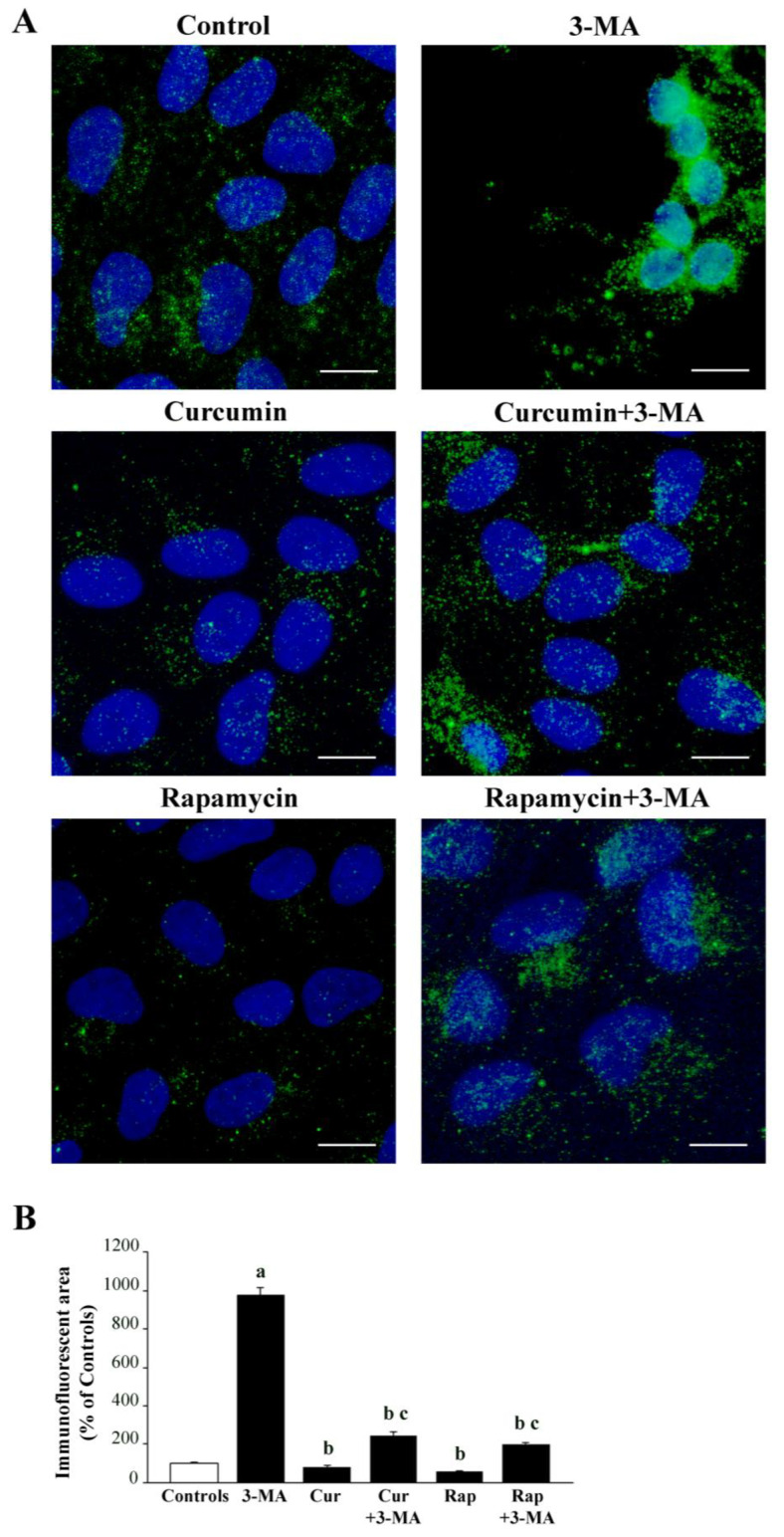
Effects of 3-MA, curcumin, or rapamycin on PS6K, the downstream product of mTOR. (**A**) Representative pictures showing the PS6K immunofluorescence and (**B**) graph reporting the PS6K immunofluorescent area, which increases following 3-MA, while it decreases following either curcumin or rapamycin when administered alone. Co-administration of curcumin or rapamycin with 3-MA counteracts the 3-MA-induced PS6K immunofluorescence. Values are expressed as the mean percentage ± S.E.M. of PS6K immunofluorescent area measured in N = 60 cells per group from three independent experiments (assuming controls = 100%). Cur = curcumin; Rap = rapamycin. ^a^ *p* < 0.05 compared with all other groups; ^b^ *p* < 0.05 compared with 3-MA; ^c^ *p* < 0.05 compared with controls. Scale bars = 20 μm.

**Figure 12:**
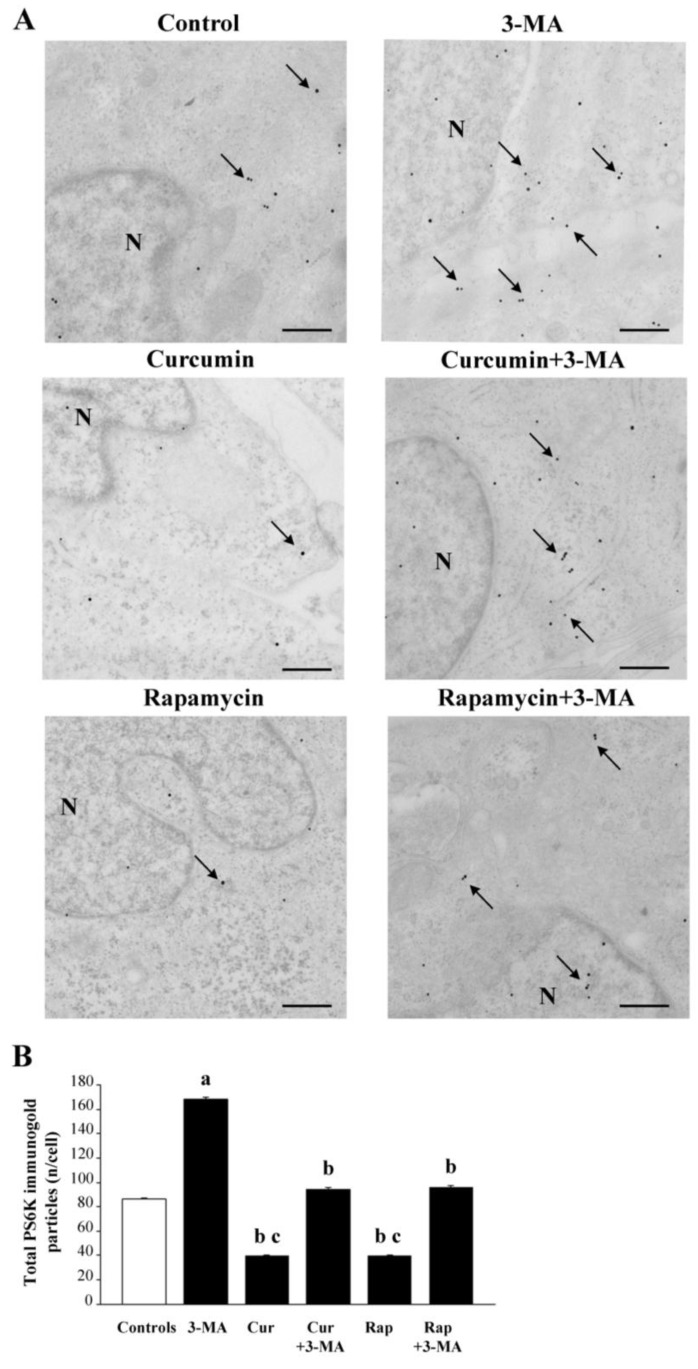
In situ stoichiometry of the downstream mTOR product PS6K. (**A**) Representative micrographs showing PS6K-immunogold particles (arrows) in RPE cells from control, 3-MA, curcumin, rapamycin, 3-MA+curcumin and 3-MA+rapamycin experimental groups. (**B**) The graph reports the count of total PS6K immunogold particles from the same experimental groups. Values are expressed as the mean ± S.E.M. measured in N = 30 cells per group. Cur = curcumin; N = nucleus; Rap = rapamycin. ^a^ *p* < 0.05 compared with all other groups; ^b^ *p* < 0.05 compared with 3-MA; ^c^ *p* < 0.05 compared with controls. Scale bars = 166 nm.

The present data, obtained following administration of 3-MA, alone or in combination with curcumin or rapamycin, are consistent with the strong modulation of these compounds on mTOR and autophagy [13,70,128,129,130,131,132,133,134,135,136,137,138]. This was measured in the following experiments carried out to confirm the tight relationship between autophagy activation and mTOR inhibition [139,140]. In this experiment, we actually measured mTOR activity by assessing the downstream enzymatic product of mTOR, PS6K, both at immunofluorescence (Figure 11) and quantitative in situ stoichiometry (Figure 12). When observing representative pictures of Figure 11A and the graph of Figure 11B, it turns out that all these compounds act on mTOR activity either as activators (3-MA) or inhibitors (curcumin or rapamycin). Therefore, the primary effect generating autophagy activation may occur directly as a consequence of mTOR modulation.

### 2.3. Quantification of mTOR and Autophagy Activity Through In Situ Stoichiometry

**Figure 13:**
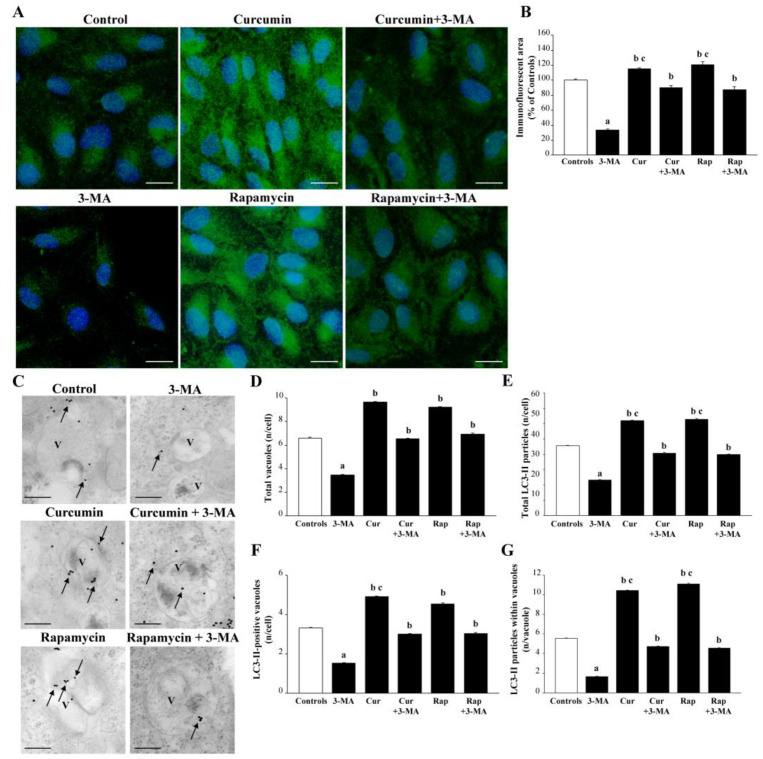
Modulation of lipidated LC3 (LC3-II) at immunostaining and in situ stoichiometry. (**A**) Representative pictures show the green immunofluorescence staining for the LC3-II isoform following various treatments, which is consistent with autophagy inhibition produced by 3-MA and its modulation induced by concomitant administration of either curcumin or rapamycin, which, when administered alone, increase the immunofluorescence above the levels measured in controls. (**B**) The graph reports the semi-quantitative measurements of LC3-II immunofluorescent areas. (**C**) Representative ultrastructural images showing the detection by immunogold of the LC3-II protein (arrows) within specific autophagy-related compartment particles. The quantitative stoichiometric measurement of LC3-II is reported in the graphs (**D**–**G**). In detail, total autophagy vacuoles (**D**), total LC3-II particles (**E**), the number of LC3-II-positive vacuoles (**F**), and the number of LC3-II protein within each vacuole (**G**) are reported. Cur = curcumin; Rap = rapamycin; V = vacuole. ^a^ *p* < 0.05 compared with all other groups; ^b^ *p* < 0.05 compared with 3-MA; ^c^ *p* < 0.05 compared with controls. Scale bars = (**A**) 20 mm; (**C**) 166 nm.

In order to better express the amount of mTOR modulation in baseline conditions and following drug administration, semi-quantitative measurements were further implemented by molecular quantification. At this aim, we added absolute quantitative assessment to semi-quantitative densitometry already reported following immunofluorescence (Figure 11), which is comparable to the semi-quantitative densitometry, which would be produced through Western blotting. In order to strengthen the quantitative and direct measurement of mTOR activity, the amount of enzyme product, PS6K, was rather provided through in situ stoichiometry within specific RPE compartments (Figure 12). Again, to cope mTOR modulation with autophagy status according to quantitative approaches, semi-quantitative immunofluorescence for the microtubule-associated protein 1A/1B-light chain 3 (LC3) type II (LC3-II) was implemented by direct visualization and counts of LC3-II protein amount within authentic autophagy-related structures, which altogether provide the ultimate and direct quantitative evidence concerning both mTOR and autophagy activities (Figure 13). These data are reported in Figure 12, where representative immunogold for PS6K confirms the suppression of the enzyme product following curcumin/rapamycin and its increase following 3-MA (representative pictures of Figure 12A and graph of Figure 12B). These data also allow the direct discernment of the cell compartments where PS6K occurs, which appears to be widespread in the cell, including the nucleus (Figure 12A).

The measurement of the specific lipidated autophagy protein LC3-II (observed at immunofluorescence and reported in representative pictures of Figure 13A) was semi-quantified in the graph of Figure 13B, and it was consistent with quantitative data about in situ stoichiometry of the very same protein (representative images of Figure 13C), within specific autophagy structures (graphs of Figure 13D–G). In these graphs, total autophagy vacuoles (Figure 13D) were counted along with total LC3-II particles (Figure 13E), the number of LC3-II-positive vacuoles (Figure 13F), and the number of LC3-II protein within each vacuole (Figure 13G). These data provide the ultimate evidence to quantify both mTOR and autophagy activity.

It remains elusive for the specific aims of the present study to search for additional potential mechanisms that might activate autophagy, aside from mTOR. Since one may assume that the present results are simply generated by the modulation of mTOR activity.

## 3. Discussion

In the present study, we profited from a quite stable cell line, which reduces experimental variables, to analyze the effects of mTOR modulation on cell viability and mitochondrial status within RPE cells. The RPE is markedly and early affected in the course of AMD, when mTOR is greatly altered [13,44,47,48,141]. Since autophagy activity is modulated by mTOR, the present study assesses mitochondrial alterations and cell viability of RPE following administration of compounds that alter mTOR. Therefore, we assessed the effects of mTOR modulation on the survival of RPE cells in relation to mitochondrial status, mTOR activity, and autophagy. In detail, we found that 3-MA is a powerful mTOR activator that produces a dose-dependent cell damage, which is rescued dose-dependently by the mTOR inhibitors curcumin or rapamycin. These effects are reciprocated by opposite variations in the autophagy status. The amount of cell damage of RPE cells was measured by using the histochemistry for H&E, the gold standard to visualize whole cell pathology, as recently further validated by using combined third-harmonic generation and three-photon fluorescence microscopy [142], without the bias of specific molecular markers. It is also confirmed by routinely assessed cell pathology [143]. This was associated with membrane-dependent permeability assay. Specifically, the cell staining following exposure with the DNA-binding compound propidium iodide, replicates data obtained by counting cell viability with classic H&E staining, although this method provides less information compared with H&E, since it is an indirect procedure to assess cell pathology. Indeed, the propidium iodide assay provides information concerning the disruption of membrane impermeability, which often anticipates cell death. Cell pathology was further assessed by measuring the amount and placement of ZO-1, a protein that confers phenotype specificity to RPE. In fact, in the course of AMD, degeneration of RPE cells is anticipated by the dismantling of ZO-1 from the cell membrane. As we reported in the conceptual images and graphs of Figure 3, the occurrence of RPE degeneration was early evident by a loss of expression of ZO-1 in the periphery of the cell, just beneath the cell membrane to stain the cell perimeter as a sort of polygonal contour. The activation of mTOR and autophagy inhibition produces a total collapse of peripheral protein expression, while the perinuclear amount was much less reduced (representative Figure 3A and graph of Figure 3B). This is in line with the findings that, in AMD, the displacement of ZO-1 more than its net decrease in the whole cell is typical [144,145,146,147,148,149,150,151,152]. This phenomenon was shown here to be modulated by mTOR along with RPE cell viability. The significance of a loss of ZO-1 from the RPE cell membrane is seminal since ZO-1 participates in the structure of tight junctions, which create the blood retina membrane [145,147,148]. This suggests that, when mTOR is activated, the ZO-1 protein is less abundant, although the main consequence concerns the subcellular placement since 3-MA fixes the protein in the perinuclear position. In contrast, in control conditions, the protein placement spreads from the nucleus to the cell membrane, being very evident on the cell contour. This is why the measurement of the total loss of ZO-1 from the cell culture does not provide effective information since (i) it is mitigated by some persistence of the perinuclear and cytosolic protein; (ii) it is not representative of the functional loss of tight junctions (which are directly related to the loss of peri-membranous ZO-1). The present study indicates that the measurement of protein amount in the whole cell does not provide the specific information related to AMD pathology, which rather concerns the perimembranous protein loss. This is why site-specific loss of optical density was established within three different areas: the perinuclear Area I; the intermediate Area II; the peri-membranous Area III. The loss of peri-membranous ZO-1 is routinely considered a hallmark to detect RPE cell degeneration. The present study indicates that the amount and spatial distribution of ZO-1 are related to cell viability counted by H&E. The cell loss was based both on actual cell count and, indirectly, the decrease in those morphological features conferring cell viability to RPE. The highest dose of 3-MA (20 mM) reduced the cell count down to 20% of controls, whereas the dose of 10 mM produced less drastic effects (cell count roughly 40% of controls). Nonetheless, many cells possess a degenerating feature and a loss (50%) of the ZO-1 protein. This indicates that the loss of peri-membranous ZO-1 is a very sensitive marker of RPE pathology, especially when considering the amount of ZO-1 placed close to the cell membrane, which is fully suppressed by the lower dose of 3-MA. This further validates the routine use of ZO-1 as a tool to assess early stages of RPE degeneration when modeling AMD [145,146,147,148,150,151]. When mTOR is activated by 3-MA, RPE cell viability may be rescued by curcumin or rapamycin, which produce a parallel increase in ZO-1 protein. The inhibition of mTOR seems to be important for the integrity of RPE since either rapamycin or curcumin is effective in counteracting 3-MA-induced cell loss at different doses, starting from 1 μM and 10 nM (for curcumin and rapamycin, respectively). The full protection was achieved by 10 μM and 100 nM of curcumin and rapamycin, respectively. These doses were extremely effective in preventing the loss of ZO-1 induced by 3-MA and possessed a significant effect in inducing ZO-1 above the levels measured in controls. This effect includes the elective placement of ZO-1 underlying the plasma membrane.

The effects of various treatments on RPE cell viability were paralleled by the effects on mitochondrial status. In detail, the activation of mTOR, which is measured following 10 mM 3-MA administration, was associated with a decrease in the whole mitochondrial mass, which is detected by MTR-G histofluorescence. The combined administration of curcumin or rapamycin, while occluding 3-MA-induced mTOR activation, prevents the decrease in the total mitochondrial mass, which is comparable with that of controls. The significance of these effects needs to be considered in the light of the specific target of the dye MTR-G, which stains mitochondria independently of their structural and functional integrity [109,110,111,112]. Therefore, the mitochondrial mass was also stained with MTR-R, which stains electively the structurally well-formed and mostly functionally effective mitochondria [113,114,115,116]. The mitochondrial mass stained with MTR-R was significantly suppressed by 10 mM 3-MA along with mTOR activation. This indicates that the loss of cell viability induced by mTOR activation was sustained by a detrimental influence on the mitochondrial status of RPE. It is remarkable that curcumin or rapamycin, when administered alone, produced a significant increase in the mitochondrial mass stained with MTR-R, which contrasts with the absence of any effects of these compounds when stained with MTR-G. The lack of effects of curcumin or rapamycin on the MTR-G-stained mitochondria contrasts with the significant effects on MTR-R-stained mitochondria, suggesting that both curcumin and rapamycin are not critical in sustaining mitochondrial removal but elevate healthy mitochondria likely due to the stimulation of the biogenesis of mitochondria. This is in line with previous studies showing the biogenesis of mitochondria following curcumin or rapamycin administration [70,153,154,155,156,157,158].

In most cell lines, the mTOR suppression is associated with a dual effect on the mitochondrial turnover consisting of both increasing the mitochondrial removal and the biogenesis of novel mitochondria [70,159,160,161,162,163,164,165,166,167,168,169,170,171,172,173,174,175,176]. In RPE cells, the mTOR-dependent biogenesis of mitochondria surpasses the mitochondrial removal. Only this condition may explain the effects of 3-MA, curcumin, or rapamycin administered alone on MTR-G and MTR-R histofluorescence. When rapamycin or curcumin is administered in combination with 3-MA, the suppression of the healthy mitochondrial mass is occluded. This suggests that mitochondrial removal may be partly independent of the mTOR pathway. This hypothesis is strengthened by the partial recruitment of the PINK1/parkin pathway in the mitochondrial turnover of these cells. This is in line with the present study and recent findings showing a relevant role of beclin1/AMBRA in regulating mitochondrial removal in RPE cells [177,178,179,180,181]. This is why, in a concomitant study, apart from sole mitochondria, we are analyzing the kind of metabolic effects that are recruited by mTOR in RPE cells. The findings discussed so far are consistent throughout the whole study we report here. In fact, when Tomm20 expression is analyzed, the data replicate what was reported for MTR-G. On one side, this lends substance to the concept that Tomm20 is indeed a marker of intrinsic mitochondrial protein, which is independent of mitochondrial integrity since Tomm20 expression was moderately decreased by 3-MA as much as MTR-G, while healthy mitochondria collapsed as witnessed by MTR-R. This correspondence between Tomm20 and MTR-G validates the significance of Tomm20 as an index of whole mitochondria mass independently of mitochondrial integrity.

A gold-standard confirmation is provided by the count of mitochondria at TEM, which was reduced when mTOR was activated. In fact, ultrastructural morphometry provides quantitative evidence indicating that 3-MA alters mitochondrial size and shape, which can be rescued by the co-administration of either curcumin or rapamycin.

Mitochondrial dynamics is strongly dependent on autophagy activation. When autophagy mitophagy is activated, mitochondrial biogenesis is enhanced [70,161,182,183,184].

This occurs through a variety of signaling pathways, which include the following: (i) Mitochondrial transcription factor A (TFAM), which promotes mitochondrial DNA replication and transcription [185,186,187]. (ii) Peroxisome-proliferator-activated receptor coactivator (PGC)-1alpha [188], which activates genes involved in mitochondrial biogenesis, including TFAM [189,190]. (iii) AMP-activated protein kinase (AMPK), which further activates PGC-1alpha [191,192,193].

The findings obtained with Tomm20 confirm that, within RPE cells, mTOR-dependent mitochondrial degradation is less pronounced than mitochondrial biogenesis, and proteolysis is not pronounced, leaving the epitopes of mitochondrial proteins similar to controls. This would not be the case in the presence of proteophagy.

In a recent overview [44], the occurrence of a number of mTOR-dependent pathways was analyzed, including various subtypes of autophagy, such as lipophagy and glicophagy, which were shown to be prevalent compared with protein degradation within RPE cells. The case of Tomm20 is emphasized by showing the merging of Pink1 with Tomm20. Pink1 acts as a fairly specific sensor of mitochondrial damage. This is confirmed by the fact that Pink1 immunostaining is increased following 3-MA. Tomm20 is reduced following 3-MA, though Pink1-positive areas merge with Tomm20-stained structures. This confirms that Pink1 specifically stains mitochondria and suggests that mitochondrial damage does not impair Tomm20 immunostaining. In line with this, Pink1 is markedly reduced following curcumin or rapamycin alone, while Tomm20 is not affected. Consistently, the merging between Tomm20 and Pink1 is reduced following curcumin or rapamycin since Tomm20 mitochondrial staining mainly concerns healthy mitochondrial regions where Pink1 is not expressed. The role of Pink1 expression at the mitochondrial level as a sensor of mitochondrial damage is addressed by the merging with its classic interactor, Parkin. In fact, Pink1-stained mitochondria are addressed to autophagy or proteasome clearance via Parkin [117,118,119,120,121,122,123,124,125].

This is in line with the recent findings showing the greatest merging of Pink1 and Parkin occurring following 3-MA. In other treatment groups, the lack of significant mitochondrial alterations abolished such a strong co-localization between these two proteins.

It needs to be mentioned that, even considering the 3-MA group, the merging between Pink1 and Parkin did not reach the extent of Pink1 and Tomm20 merging since the removal of altered mitochondria within the RPE cells occurs only partially via the classic autophagy-dependent Pink1/Parkin interaction, and it occurs via exploiting other proteins, such as Beclin1 and AMBRA1 [181,194,195]. The net effects of these treatments concerning the mitochondrial status were confirmed in the present study by ultrastructural morphometry, which indicates how the inferences made on the basis of light microscopy data are correct. When comparing the values for total and altered mitochondria in each treatment group analyzed at TEM, it is evident that the decrease in total mitochondria produced by 3-MA is mainly due to a lack of mitochondrial renewal. In fact, despite 3-MA producing a decrease in total mitochondria, altered mitochondria are twofold compared with controls. In contrast, when curcumin or rapamycin is administered, the total number of mitochondria does not differ noticeably from controls, although the amount of healthy mitochondria is greatly increased (reciprocated by altered mitochondria). Thus, it seems that the decrease in total mitochondria observed following 3-MA is due to a collapse in mitochondria biogenesis rather than an accelerated mitochondrial removal.

Altogether, these data need to be transferred to the pathological condition of AMD, where a strong autophagy inhibition within RPE cells is thought to occur [15,126]. Therefore, it is not surprising that, in AMD, just like within 3-MA administered RPE cells, mitochondrial alterations are prominent. However, one may expect that the outcome of mTOR activation/autophagy inhibition would lead to the accumulation of stagnant altered mitochondria, with an excess of mitochondrial mass, as described following mitochondrial toxins [70,196]. Surprisingly, here we found a net decrease in total mitochondria, which reciprocates analogous findings from RPE cells within AMD patients. Such a discrepancy is fascinating and a witnesses to specific alternative phenomena. Thus, the accumulation of non-digested Tomm20-positive, Pink1-positive, MTR-G-stained mitochondria is not overwhelming compared with other mechanisms of cell pathology within AMD-RPE cells. It is well known that in most cells and areas of the CNS mitochondrial removal goes along with the biogenesis of novel mitochondria, following a harmonic pattern. This is further confirmed by the findings that mitochondrial biogenesis is associated with autophagy activation, since these biochemical pathways are interconnected.

The parallel effects on mitophagy and mitochondriogenesis were reported by Palikaras and Tavernakis [182], who showed the harmonic relationship between mitochondrial removal and mitochondrial renewal in various areas [160,161,162,163,165,197], including the retina [198].

In mammalian cells, mitophagy mainly occurs through activation of the Pink1/Parkin pathway. These signaling pathways concomitantly stimulate the coordinated induction of mitochondrial biogenesis. The imbalance of these activities, as apparent in the present study, is expected to lead to an excess of mitophagy in the absence of mitochondriogenesis [198]. This suggests that mitophagy is also triggered via pathways other than Pink1/Parkin. For instance, AMBRA1 interaction with Beclin1 may lead to mitochondrial removal in the absence of mitochondrial biogenesis. It looks like that in AMD, just like what is found in the course of CNS degenerative disorders such as Parkinson’s disease (PD) and Alzheimer’s disease (AD) [177,178,180,181], there is a loss of orchestration between mitochondriogenesis and mitophagy. This seems to be typical of RPE cells, where mTOR activation produces only a moderate suppression of mitochondrial removal, while mitochondrial biogenesis is markedly affected.

This is likely to depend on the pivotal role of AMBRA1 in regulating mitochondrial turnover within RPE cells [181]. In fact, as elegantly reported, AMBRA1 may promote mitophagy, either by regulating the Pink1/Parkin pathway or following a Pink1-independent pathway [181]. This explains why, in AMD patients, as well as in the present study within RPE cells, Baseline levels of Pink1 are very low [199].

The progressive damage likely leads to a net decrease in mitochondria, where the remaining organelles, despite being reduced, possess severe alterations.

Finally, to cope mTOR modulation with autophagy status we measured the mTOR downstream enzyme product, PS6K, as well as the specific lipidated autophagy protein LC3-II through quantitative in situ stoichiometry, which was added to semi-quantitative immunofluorescence. We found that the suppression of PS6K following curcumin/rapamycin and its increase following 3-MA, occurs in concomitance with an increase in the amount of LC3-II within specific autophagy structures following treatments with curcumin/rapamycin and its decrease following 3-MA. Altogether these findings confirm through a direct quantitative evidence, the link between mTOR modulation and autophagy [50,51,53,56,57].

## 4. Materials and Methods

### 4.1. Cell Culture and Treatments (The Basis of the Experimental Design)

The Adult Retinal Pigment Epithelial cell line-19 (ARPE-19), obtained from a cell bank (Bio-banking of Veterinary Resources, Brescia, Italy), is a spontaneously growing human cell line derived from the normal eyes of a 19-year-old male. Cells were grown in accordance with the provider’s technical data sheet. Specifically, the culture medium consisted of Dulbecco’s modified Eagle’s medium (DMEM, Sigma-Aldrich, St. Louis, MO, USA) and Nutrient Mixture F12 (Sigma-Aldrich), 1:1 mixture, supplemented with 20% fetal bovine serum (FBS, Sigma-Aldrich) and penicillin (50 IU/mL)/streptomycin (50 mg/mL, Sigma-Aldrich), under standard culture conditions (5% CO_2_ at 37 °C). At the time of the experiments, cells were seeded into culture plates, incubated at 37 °C in 5% CO_2_ for 24 h. In detail, for Transmission Electron Microscopy (TEM), 1 × 10^6^ ARPE-19 cells were seeded in culture dishes to reach a final volume of 5 mL. For light microscopy experiments, 2 × 10^4^ cells were seeded on slides placed in 24-well plates to reach a final volume of 1 mL/well. For viability assay with propidium iodide 4 × 10^4^ cells were seeded in 24-well plates in a final volume of 1 mL/well.

ARPE-19 cells were treated with the classic 3-MA (Sigma-Aldrich, 10 mM and 20 mM) dissolved in pre-warmed (37 °C) culture medium for 72 h. These cells were compared with culture dishes (Controls), where ARPE-19 received only culture medium (same volume, same temperature). The effects produced by 3-MA alone on cell viability were tentatively antagonized by various doses of the classic mTOR inhibitor rapamycin (Sigma-Aldrich) and various doses of the phytochemical curcumin (Sigma-Aldrich) [13,102,200]. In detail, both rapamycin and curcumin were tested for their neuroprotective effects by carrying out a dose–response study, which was designed based on pilot experiments, where the doses of rapamycin were roughly shifted 10^2^ to the left on the X axis compared with the dose–response curve for curcumin. Therefore, to assess cell viability, the dose–response experiments consisted of administering ARPE-19 with different doses of curcumin (0.1 μM, 1 μM, and 10 μM) or rapamycin (1 nM, 10 nM, and 100 nM) alone or in combination with 3-MA (10 mM and 20 mM) for 72 h (see also Table 1). Stock solutions of curcumin, rapamycin, and 3-MA were prepared. In detail, a stock solution of curcumin 10 mM was obtained by dissolving 3.68 mg of curcumin powder in 1 mL of DMSO (Sigma-Aldrich), while the doses of rapamycin were prepared starting from a stock solution of 1 mM. This was obtained by dissolving 0.91 mg rapamycin powder in 1 mL of a solution of culture medium containing 10% DMSO. A stock solution of 3-MA 100 mM was obtained by dissolving 14.91 mg of 3-MA powder in 1 mL of the pre-warmed culture medium. Final concentrations of curcumin, rapamycin, and 3-MA being used for each treatment were obtained by diluting appropriate aliquots of the stock solutions within the cell culture medium. When added in combination with 3-MA, curcumin or rapamycin was administered 2 h before 3-MA administration. The range of doses was based on our previous studies on the effects of 3-MA in other cell lines [91] or assessed specifically in ARPE, as previously reported [13]. The dose–response experiments were seminal to assessing the efficacy of mTOR inhibition and/or stimulation in modulating cell viability. Again, these studies allowed us to plan an experimental approach aimed at modulating mTOR to an extent that is still compatible with cell survival. This was expected to provide insight into the persisting effects of mTOR activation on RPE cell pathology. In this way, the study of mitochondrial status, as well as plain cell viability and the loss of tight junctions through the ZO-1 protein expression, could be appropriately designed and correlated. This first set of experiments allowed us to select the doses of rapamycin (100 nM), curcumin (10 μM), and 3-MA (10 mM) to be used to investigate the effects of mTOR modulation on the development of AMD-like phenotype.

### 4.2. Cell Viability

#### 4.2.1. H&E

Cells were fixed with 4% paraformaldehyde for 15 min, washed in PBS, and then immersed in the hematoxylin solution (Sigma-Aldrich) for a few minutes. After stopping the hematoxylin staining through repeated washing in running water, cells were plunged into eosin solution (Sigma-Aldrich) for a few seconds and washed out again to remove the excess of dye. Cells were dehydrated by increasing alcohol solutions, clarified in xylene, and finally covered with DPX mounting medium (Sigma-Aldrich). Stained cells were observed under a Nikon Eclipse Ni light microscope (Nikon, Tokyo, Japan), equipped with a digital camera connected to the NIS Elements software (NIS Elements D 5.30.00-build 1531-64-bit) for image analysis. H&E-stained cells were counted under 20× magnification within three microscopic fields, where only distinct cells that were not overlapping could be detected. Values are given as the mean percentage ± S.E.M. (assuming controls as 100%) from three independent experiments. Data are compared using ANOVA with Scheffe’s post hoc test. Differences between the various groups are considered to be significant when the null hypothesis H_0_ is less than 5%.

#### 4.2.2. Propidium Iodide Viability Assay

At the end of the treatments, both floating and adherent cells were harvested and, after staining with 2 µg/mL propidium iodide (P1304MP, Thermo Fisher Scientific, Waltham, MA, USA) for 5 min, they were analyzed using an ACCURI C6 PLUS (BD Biosciences, Franklin Lakes, NJ, USA) flow cytometer. Doublet discrimination was manually carried out using a linear scale where events were plotted based on FSC signal area versus FSC signal height. Gated events were then visualized on a linear scale based on FSC signal area versus 585/40 FL2 channel signal area in a logarithmic scale, in order to create a plot distinguishing dead (red) cells from live (blue) cells. Data refer to three independent experiments, with a minimum of n = 2500 events per experimental group. Data are given as the mean ± S.E.M. percentage of propidium iodide-positive cells out of the total cells. Data are compared using ANOVA with Scheffe’s post hoc test. Differences between the various groups are considered to be significant when the null hypothesis H_0_ is less than 5%.

### 4.3. Mitochondrial Histofluorescence

#### 4.3.1. MitoTracker Green and MitoTracker Red

To stain mitochondria in living cells, 5 × 10^4^ cells were grown in 24-well plates containing 1 mL/well of culture medium. At the end of the treatments, the medium was removed, and cells were incubated in a solution of MTR-G (Thermo-Fisher Scientific), which labels total (both healthy and degenerating) cell mitochondria, or MTR-R (Thermo-Fisher Scientific), which reveals healthy mitochondria [111,201,202]. Both fluorescent dyes were used at 500 nM (Table 1) in a serum-free culture medium for 45 min, at 37 °C and 5% CO_2_. At the end of the incubation, the staining solutions were removed, fresh pre-warmed medium was added, and cells were immediately analyzed by fluorescence microscopy (Nikon).

The optical density was measured under a fluorescence microscope using ImageJ software (NIH, Version 1.8.0_172, Bethesda, MD, USA) according to Marwaha and Sharma [129]. Values are given as the mean percentage ± S.E.M. of fluorescent areas per cell, N = 60 cells/group (assuming the mean in control cells as 100%). The data refer to three independent experiments. In this procedure, the intensity of histofluorescence, which peaked within specific spots, was not considered since it may depend on variation in mitochondrial structure, which could not be addressed by this method and was further quantified at TEM. On the other hand, these peaks were minimal, and fluorescent areas were quite uniform in intensity.

Data are compared using ANOVA with Scheffe’s post hoc test. Differences between the various groups are considered to be significant when the null hypothesis H_0_ is less than 5%.

#### 4.3.2. Immunofluorescence

At the end of treatments, the medium was removed, and cells were washed in PBS and fixed with 4% paraformaldehyde in PBS for 15 min. After exposure to 0.1% TritonX-100 in PBS for 15 min, cells were plunged in 10% normal goat serum in PBS for 1 h at room temperature. Then, cells were incubated overnight at 4 °C in a PBS solution containing the primary antibodies and 1% normal goat serum. The following primary antibodies were used (Table 1):

(i) Rabbit anti-Parkin (Sigma-Aldrich); (ii) rat anti-ZO-1 (Sigma-Aldrich); (iii) mouse anti-Pink1 (Abcam, Cambridge, UK); (iv) rabbit anti-Tomm20 (Abcam); (v) mouse anti-PS6K (Cell Signaling, Danvers, MA, USA); (vi) mouse anti-LC3-II (Cosmo Bio Inc., Carlsbad, CA, USA). All primary antibodies were diluted 1:100, except for the anti-PS6K primary antibody, which was diluted 1:50.

Cells were rinsed in PBS and incubated for 1 h and 30 min with the appropriate fluorophore-conjugated secondary antibodies (Table 1), Alexa488 (green fluorophore, Life Technologies, Carlsbad, CA, USA) or Alexa546 (red fluorophore, Life Technologies). All secondary antibodies were diluted 1:200 in PBS. To stain cell nuclei, slides were washed in PBS, and then they were immersed for 5 min in an aqueous solution of the fluorescent dye DAPI (1:1000, Sigma-Aldrich, Table 1). Finally, cell slides were washed in PBS, gently transferred to a coverslip, and covered with the mounting medium Fluoroshield (Sigma-Aldrich).

Slides were observed using a Nikon Eclipse Ni light microscope, which was equipped with a fluorescent lamp and a digital camera connected to NIS Elements software (NIS Elements D 5.30.00-build 1531-64-bit) for image analysis (Nikon). All experiments were carried out in triplicate. Control slides were incubated with secondary antibodies only.

The measurement of fluorescence (either following histofluorescence staining or immunohistofluorescence) was based on a cut-off of intensity according to Marwaha and Sharma [203]. Immunofluorescent areas were measured using the software ImageJ (NIH, Version 1.8.0_172, Bethesda, MD, USA). In detail, arbitrary amounts of cell fluorescence were calculated according to Marwaha and Sharma [203], the mean fluorescent area per cell was calculated and reported as a semi-quantitative measurement in specific graphs. Values are given as the mean percentage ± S.E.M. of immunofluorescent area (assuming controls = 100%) measured in N = 60 cells per experimental group.

Merging areas of immunofluorescence were measured in μm^2^ using ImageJ software (NIH, Version 1.8.0_172, Bethesda, MD, USA), and values are given as the mean percentage of controls merging areas ± S.E.M. per cell from N = 60 cells per group (assuming controls = 100%). Data are compared using ANOVA with Scheffe’s post hoc test.

Differences between the various groups are considered to be significant when the null hypothesis H_0_ is less than 5%.

Finally, ZO-1 immunofluorescence was expressed both as a fluorescent area and linear extent along the cell contour as calculated using ImageJ software (NIH, Version 1.8.0_172, Bethesda, MD, USA). Area was calculated in μm^2^ and values are given as the mean percentage ± S.E.M. of immunofluorescent area (assuming controls = 100%). Instead, linear extent was calculated and expressed in μm and reported as the mean length ± S.E.M. per cell from N = 60 cells per group. Data are compared using ANOVA with Scheffe’s post hoc test. Differences between the various groups are considered to be significant when the null hypothesis H0 is less than 5%.

All light microscopy data were calculated from three independent experiments, and the means were further considered.

Significance of each antibody used. In these experiments, primary antibodies were selected to investigate the integrity of intercellular tight junction (ZO-1), the mitochondrial integrity (Tomm20), and biochemical pathways in RPE cells related to mitochondrial turnover (Pink1 and Parkin), which are involved in the disease course of AMD. The prominent role of mTOR as an enzyme complex with hegemonic control on mitochondria and the autophagy pathway was investigated by measuring the downstream product of mTOR activity, PS6K. This corresponds to a mitogen-activated Ser/Thr protein kinase. PS6K phosphorylates the S6 protein of the 40S ribosomal subunit, and it is involved in the translational control of 5′ oligopyrimidine tract mRNAs. Thus, by measuring the staining of PS6K, it is possible to have an estimate of mTORC1 activity [204,205].

In this way, altered mitochondrial imaging observed at histofluorescence through the staining of total (MTR-G) and healthy (MTR-R) mitochondria was significantly strengthened by the immunostaining against the constitutive mitochondrial protein Tomm20, which provides the transfer of proteins from the cytosol to mitochondria. Similarly, the mitochondrial status was investigated by combining Tomm20 with Pink1, since the latter antigen is expressed on the mitochondrial structure upon mitochondrial alterations [121]. The staining for Pink1 was also combined with its natural interactor (Parkin), which addresses altered mitochondria towards proteasome and/or autophagy degradation (mitophagy). The specific loss of tight junction proteins such as ZO-1 was investigated in light of its involvement in AMD. In this way, histochemistry was combined with specific immunostaining in order to obtain reliable information on cell viability, mitochondrial status, and actual mTOR activity following mTOR activators or inhibitors. These data provided a rough imaging of RPE cells, which were further detailed by in-depth morphology by using electron microscopy.

### 4.4. Transmission Electron Microscopy

Adherent RPE cells were directly fixed on culture dishes, following replacement of culture medium with a solution of 2% paraformaldehyde and 0.1% glutaraldehyde. Fixed cells were then (90 min after) scraped from the dish and collected in a tube to be centrifuged at 10,000× *g* for 5 min. The fixing solution containing two aldehydes was dissolved in phosphate-buffered saline, and it was selected to provide optimal preservation of cellular architecture while ensuring minimal masking of antigenic epitopes [91,206,207,208].

The fixing aldehyde solution has a pH of 7.4 and interacts with cell structures for 90 min, at 4 °C. Following fixing, samples were further exposed to a PBS solution containing 1% (in volume) osmium tetroxide (OsO_4_) to undergo further fixing for one hour at 4 °C.

Following OsO_4_, pellets were dehydrated in scalar graded ethanol solutions to be further plunged in a propylene oxide solution, which enables sample permeation by epoxy resin. In fact, a 1:1 solution containing propylene oxide and epoxy resin interacted with the sample overnight. The day after, pure epoxy resin replaces the previous solution three times at 40 min intervals (20 min at room temperature and 20 min at 60 °C). Following intermittent heating cycles, the samples were plunged in pure epoxy resin for 72 h (60 °C). These time intervals allow resin polymerization once the resin has fully permeated the sample’s trim. This stage led the resin block to form a whole with the specimen, allowing consistency and shape to be finally cut at an ultra-microtome (and stored for additional purposes). When TEM analysis required a general sample view, the previous step was carried out at lower magnification to cut semi-thin slices (1 μm thick), which were stained with toluidine blue. Ultra-thin slices (90 nm thick) were used for plain electron microscopy. The ultra-microtome (Power Tome, RMC Products, Boeckler Instruments, Tucson, AZ, USA) served both for semi-thin and ultra-thin slices.

Ultra-thin slices were layered on grids to be further processed by using uranyl acetate, followed by lead citrate. Analysis of grids containing ultra-thin slices was carried out by using a Jeol JEM 100SX TEM from Jeol (Tokyo, Japan).

### 4.5. Post-Embedding Immuno-Electron Microscopy

Ultra-thin slices were collected on nickel grids, de-osmicated in aqueous solution saturated by sodium metaperiodate (NaIO_4_) for 15 min, and then washed three times for 10 min in ice-cold filtered PBS (pH 7.4). Grids were first immersed in ice-cold PBS containing 10% normal goat serum and 0.2% saponin for 20 min at room temperature. Then, they were incubated within a solution, containing anti-PS6K-Ab-I (Cell Signaling) diluted 1:50 (Table 1) or anti-LC3-II (Cosmo Bio Inc.) diluted 1:50 (Table 1), 1% normal goat serum and 0.2% saponin in ice-cold PBS, in a humidified chamber overnight, at 4 °C. After washing in PBS, grids were incubated within a solution containing gold-conjugated secondary antibodies (gold particle diameter 20 nm, BB International, Cardiff, UK) diluted 1:50, in PBS containing 1% normal goat serum and 0.2% saponin for 1 h, at room temperature. After rinsing in PBS, grids were incubated in 1% glutaraldehyde for 3 min, washed in distilled water, and finally stained with uranyl acetate and lead citrate. Ultra-thin sections were observed at Jeol JEM 100SX TEM from Jeol (Tokyo, Japan). Control sections were incubated with secondary antibodies only.

The number of total PS6K or LC3-II immuno-gold particles per cell and the number of LC3-II particles within vacuoles were expressed as the mean ± S.E.M. from N = 30 cells per experimental group. Data are compared using ANOVA with Scheffe’s post hoc test.

Differences between the various groups are considered to be significant when the null hypothesis H_0_ is less than 5%.

### 4.6. Ultrastructural Morphometry

From each grid, several slices (90 nm thick) either in serial or non-serial order were examined at 6000× magnification to assess cell pathology and mitochondrial integrity, including the count of total, healthy, and damaged mitochondria.

Mitochondrial identification was based upon the presence of inner and outer membranes, the internal matrix, and a well-shaped crests system. Despite natural morphological variations occurring under physiological conditions, mitochondrial alterations are scored by the observer on the basis of ultrastructural criteria that were validated in previous studies [121,209,210,211,212].

For mitochondrial ultrastructural morphometry, values were expressed as follows: (i) total number of mitochondria per cell; (ii) percentage of altered mitochondria per cell; (iii) mitochondrial area; (iv) mitochondrial maximum and minimum diameter; (v) mitochondrial perimeter. Values are given as the mean number or the mean percentage ± S.E.M. per cell from N = 40 cells per group (i and ii), or N = 30 cells per group (iii–v).

The number of total or LC3-II-stained cell vacuoles was also assessed and values are given as the mean number ± S.E.M. per cell from N = 30 cells per group.

Data are compared using ANOVA with Scheffe’s post hoc test. Differences between the various groups are considered to be significant when the null hypothesis H_0_ is less than 5%.

### 4.7. Detailed Statistical Analysis

#### 4.7.1. Analysis of Cell Viability

Count of H&E-stained cells. Cell count was carried out under a light microscope, at 20× magnification; the number of H&E-stained cells in each experimental group was counted and expressed as the mean percentage ± S.E.M. of the control group (which corresponds to 100%). The data refer to three independent experiments. The issue of relying on the sole H&E staining to assess cell damage by light microscopy could be debatable in terms of the lack of specific markers of neurodegeneration (as instead provided by trypan blue and Fluoro Jade-B [213]). As a matter of fact, we already combined all these procedures together and we discussed the significance of their variability, while the rough values are approximately steady. Moreover, in the present manuscript, we did provide evidence for ZO-1 immunostaining, which is routinely considered a marker for the specific integrity of the RPE monolayer and represents the gold standard to assess RPE cells’ integrity in experimental models aimed to mimic the development of AMD [214,215]. The amount of severe cell damage assessed by H&E is based on the actual lack of cell structures (unique among light microscopy procedures used here) and the presence of remarkable alterations of cell shape and cell size, and the occurrence of faint cytosol visible as pale eosinophilic cytosolic areas, along with intensely hematoxylin-stained, pyknotic nuclei. In keeping with H&E staining, when counting the amount of cell damage, we included those cells where these alterations are severe. In fact, in addition to decreased cell number, in 3-MA-treated ARPE we found pale vacuolar cytosolic areas, irregular shape, and smaller size compared with homogenously stained cytosol and regular cell shape of the controls. Consistency across different techniques expressing cell damage at light microscopy (H&E histochemistry and ZO-1) immunohistochemistry and between light and electron microscopy was remarkable concerning the amount of cell damage, and values were quite steady, which internally validates the procedures applied here.

#### 4.7.2. Analysis of Mitochondria

At light microscopy, this was based both on histochemistry (MTR-G and MTR-R) and immunohistochemistry of intrinsic mitochondrial proteins (Tomm20 immunohistochemistry) or mitochondria-associated proteins (Pink1 and Parkin immunohistochemistry). Namely, the analysis of MTR-G histochemistry provides a rough estimate of the total amount of mitochondria. This is expressed as the mean percentage of the stained area per cell, assuming the value of controls = 100%. The total number of cells was 60 per group. Since the value of MTR-G provides the amount of both healthy and altered mitochondria, this count needs to be integrated with MTR-R, which stains healthy mitochondria, to obtain a rough estimate of both total, healthy, and indirectly, fairly damaged mitochondria. In fact, both MTR-G and MTR-R bind cysteinyl groups and accumulate within the mitochondrial matrix [110]. However, the entry of MTR-G occurs constantly, independently of the transmembrane mitochondrial potential, while the entry of MTR-R is dependent on the preservation of physiological transmembrane mitochondrial potential [116]. This makes MTR-R a marker of physiological mitochondria, while MTR-G stains both healthy and damaged mitochondria [109,110,111,112,113,114,115,216,217,218,219,220,221,222,223,224,225,226,227,228]. The amount of healthy mitochondria is expressed as the mean percentage of the red-stained area per cell, assuming the value of controls = 100%. The immunostaining for Tomm20 is expected to stain a specific mitochondrial protein. Immunostaining for Tomm20 is calculated as the mean immunopositive area per cell (calculated in μm2 and expressed as the mean percentage of the immuno-stained area per cell, assuming the value of controls = 100%) from 60 cells per group. Tomm20 immunostaining and MTR-G histochemistry are quite similar in all treatment groups. In contrast, Tomm20 immunostaining is in excess of MTR-R, which suggests that mitochondrial degradation does not involve proteolysis of structural mitochondrial proteins, which are still detectable by Tomm20 immunostaining. Tomm20 immunostaining was carried out either alone or in combination with Pink1, which senses mitochondrial alterations. The amount of Pink1 positive areas is calculated as the mean immunopositive area per cell (calculated in μm^2^ and expressed as the mean percentage of the immuno-stained area per cell, assuming the value of controls = 100%) from 60 cells per group. The merge between Tomm20 and Pink1 is calculated as the mean of merged area (calculated in μm^2^ and expressed as the mean percentage of the immuno-stained area per cell, assuming the value of controls = 100%) per cell, from 60 cells per group. Since Pink1 is typically expressed at the mitochondrial level following damage and it works to sense and warn mitochondrial alterations for Parkin-mediated mitochondria removal [117], Pink1 immunostaining was also combined with Parkin immunostaining. This is based on the kind of interactions of Pink1, mostly occurring with Parkin to address mitochondria towards proteasome- and/or autophagy-mediated clearance. The merge between Pink1 and Parkin is calculated as the mean of merged immunopositive area per cell (calculated in μm^2^ and expressed as the mean percentage of the immuno-stained area per cell, assuming the value of controls = 100%) from 60 cells per group. All these data produced by light microscopy represent a rough estimate of the real number of total, altered, and healthy mitochondria being presented in the descriptive statistics. Inferential statistics for light microscopy was carried out by using ANOVA with Sheffè’s post hoc test. This statistical analysis is borderline, considering the parametric and non-parametric nature of the values being compared. In detail, the in-depth analysis of these numbers reveals the non-linear distribution and the rough nature of optical densitometry. Therefore, according to a routine procedure, ANOVA was used, still keeping the inferential conclusions under scrutiny. To validate these data obtained at light microscopy in the light of the real amount (quantitative data) we added ultrastructural morphometry to produce values according to a normal distribution, thus reporting absolute parametric quantitative data. In this case, data can be treated according to classic mathematics. The strengthening of quantitative data obtained from each group, allowing parametric inferential statistics, was achieved by the use of ultrastructural morphometry at plain TEM (for organelle counts). In the case of mitochondrial alterations, the ultrastructural morphometry was described in a total of 40 cells per group, while mitochondrial area, perimeter and maximum and minimum diameter were counted in N = 30 cells per group. Mitochondrial alterations were characterized as follows according to our previous studies [70,212]: (i) decreased matrix electron density (dilution, vacuolization, and presence of electron-lucent foci; (ii) fragmented, disorganized, and ballooned crests (intra-crest swelling); (iii) partial or complete separation of the outer and inner mitochondrial membrane; (iv) occurrence of dense, osmiophilic laminated or whorled membranes within mitochondria; (v) mitochondrial swelling.

In order to be considered in the group of altered mitochondria, a given mitochondrion was expected to carry on at least two of these alterations. Altered mitochondria were reported in each group as the mean percentage of altered mitochondria per cell, assuming N = 100, the total number of mitochondria. Comparisons between groups were carried out by ANOVA with Sheffè’s post hoc test.

## 5. Conclusions

In AMD, mitochondria are strongly affected, which is associated with pronounced sensitivity to oxidative stress and the occurrence of high amount of reactive oxygen species (ROS) [48]. This explains why within RPE cells in the course of AMD, there is a shift in energetic metabolism, where ATP production moves from mitochondrial respiratory chain to cytosolic anaerobic glycolysis, which requires a higher amount of glucose. As reported in the present study, altered mitochondria are difficult to renew [48]. These pathological mechanisms grounded on mTOR activation get worse with age, making it more likely the onset of AMD [194]. Thus, a defect in mitochondrial biogenesis may be key in the course of the disease and seems to be replicated by the stimulation of mTOR. The potential benefit of specific compounds (rapamycin or curcumin), which seem to improve mitochondrial renewal, is beneficial to relieve the mitochondrial failure affecting patients with AMD. The present findings are relevant to various mechanisms of neurodegeneration occurring in acute and chronic disorders of the CNS, such as AD [229,230], PD [231,232,233], Huntington’s disease [234,235], epilepsy [236,237], and neurodegeneration associated with brain tumors [238] or abnormal involuntary movements [239], the neuronal damage induced by psychostimulants [196] where mitochondrial alterations and mTOR dysfunction are often concomitant [240]. These considerations bring the retina back to its ontogenetic origin, which is inside the CNS. Therefore, it is not surprising that cell clearing systems are similarly involved in CNS and retinal degenerative disorders [241]. This paves the way to the beneficial effects of phytochemicals, which are emerging as powerful modulators of autophagy and mTOR [242]. In line with this, the present research study provided further evidence, which correlates autophagy an mTOR activity as powerful modulators of the mitochondrial status.

## Figures and Tables

**Table 1:** Concentrations and time of exposure of the reagents used in the study.

Reagents	Concentrations	Time of Exposure
3-methyladenine (3-MA)	10 mM	72 h
	20 mM	72 h
Curcumin	0.1 μM	72 h
	1 μM	72 h
	10 μM	72 h
Curcumin+3MA:		
Curcumin	10 μM	74 h (2 h before 3-MA)
3-MA	20 mM	72 h
Rapamycin	1 nM	72 h
	10 nM	72 h
	100 nM	72 h
Rapamycin+3-MA:		
Rapamycin	100 nM	74 h (2 h before 3-MA)
3-MA	20 mM	72 h
MitoTracker Green (MTR-G)	500 nM	45 min
MitoTracker Red (MTR-R)	500 nM	45 min
rat anti-ZO-1 Ab-I	1 μg/mL	overnight
rabbit anti-Parkin Ab-I	10 μg/mL	overnight
mouse anti-Pink1 Ab-I	10 μg/mL	overnight
rabbit anti-Tomm20 Ab-I	4.3 μg/mL	overnight
mouse anti-PS6K Ab-I	14.38 μg/mL	overnight
	(both IF and TEM-Ig)	
mouse anti-LC3-II	1 μg/mL (for IF)	overnight
	2 μg/mL (for TEM-Ig)	overnight
Alexa488 Ab-II	10 μg/mL	90 min
Alexa546 Ab-II	10 μg/mL	90 min
20 nm gold-conjugated Ab-II	3 μg/mL	1 h
DAPI	20 μg/mL	5 min

Ab-I: primary antibody; Ab-II: secondary antibody; IF: immunofluorescence; TEM-Ig: immunogold at TEM.

## Data Availability

The raw data supporting the conclusions of this article will be made available by the authors upon request.

## Supplementary Materials

The following supporting information can be downloaded at https://www.mdpi.com/article/10.3390/ijms26199442/s1.

## References

[1] Yu B., Xu P., Zhao Z., Cai J., Sternberg P., Chen Y. (2014). Subcellular distribution and activity of mechanistic target of rapamycin in aged retinal pigment epithelium. Investig. Ophthalmol. Vis. Sci..

[2] Lopes de Faria J.M., Duarte D.A., Montemurro C., Papadimitriou A., Consonni S.R., Lopes de Faria J.B. (2016). Defective Autophagy in Diabetic Retinopathy. Investig. Ophthalmol. Vis. Sci..

[3] Yao A., van Wijngaarden P. (2020). Metabolic pathways in context: mTOR signalling in the retina and optic nerve—A review. Clin. Exp. Ophthalmol..

[4] Wang Y., Fung N.S.K., Lam W.C., Lo A.C.Y. (2022). mTOR Signalling Pathway: A Potential Therapeutic Target for Ocular Neurodegenerative Diseases. Antioxidants.

[5] Markitantova Y., Simirskii V. (2025). Retinal Pigment Epithelium Under Oxidative Stress: Chaperoning Autophagy and Beyond. Int. J. Mol. Sci..

[6] Bergmann M., Schütt F., Holz F.G., Kopitz J. (2004). Inhibition of the ATP-driven proton pump in RPE lysosomes by the major lipofuscin fluorophore A2-E may contribute to the pathogenesis of age-related macular degeneration. FASEB J..

[7] Kaushal S. (2006). Effect of rapamycin on the fate of P23H opsin associated with retinitis pigmentosa (an American Ophthalmological Society thesis). Trans. Am. Ophthalmol. Soc..

[8] Kunchithapautham K., Rohrer B. (2007). Apoptosis and autophagy in photoreceptors exposed to oxidative stress. Autophagy.

[9] Mellén M.A., de la Rosa E.J., Boya P. (2008). The autophagic machinery is necessary for removal of cell corpses from the developing retinal neuroepithelium. Cell Death Differ..

[10] Ryhänen T., Hyttinen J.M., Kopitz J., Rilla K., Kuusisto E., Mannermaa E., Viiri J., Holmberg C.I., Immonen I., Meri S. (2009). Crosstalk between Hsp70 molecular chaperone, lysosomes and proteasomes in autophagy mediated proteolysis in human retinal pigment epithelial cells. J. Cell. Mol. Med..

[11] Cai J., Liao F., Mao Y., Liu S., Wu X., Tang S., Wang S., Shan G., Wu S. (2024). Regulation of LAMTOR1 by oxidative stress in retinal pigment epithelium: Implications for age-related macular degeneration pathogenesis. Exp. Eye Res..

[12] Nashine S., Cohen P., Chwa M., Lu S., Nesburn A.B., Kuppermann B.D., Kenney M.C. (2017). Humanin G (HNG) protects age-related macular degeneration (AMD) transmitochondrial ARPE-19 cybrids from mitochondrial and cellular damage. Cell Death Dis..

[13] Pinelli R., Ferrucci M., Biagioni F., Bumah V., Scaffidi E., Puglisi-Allegra S., Fornai F. (2023). Curcumin as a Perspective Protection for Retinal Pigment Epithelium during Autophagy Inhibition in the Course of Retinal Degeneration. Curr. Neuropharmacol..

[14] Ding X., Cao S., Wang Q., Du B., Lu K., Qi S., Cheng Y., Tuo Q.Z., Liang W., Lei P. (2024). DNALI1 Promotes Neurodegeneration after Traumatic Brain Injury via Inhibition of Autophagosome-Lysosome Fusion. Adv. Sci..

[15] Kaarniranta K., Blasiak J., Liton P., Boulton M., Klionsky D.J., Sinha D. (2023). Autophagy in age-related macular degeneration. Autophagy.

[16] Hyttinen J.M.T., Koskela A., Blasiak J., Kaarniranta K. (2024). Autophagy in drusen biogenesis secondary to age-related macular degeneration. Acta Ophthalmol..

[17] Wang X.L., Gao Y.X., Yuan Q.Z., Zhang M. (2024). NLRP3 and autophagy in retinal ganglion cell inflammation in age-related macular degeneration: Potential therapeutic implications. Int. J. Ophthalmol..

[18] Awad A.M., Seetharaman A.T.M., Hossain M.S., Elshaer S.L., Abdelaziz R.R., Nader M.A., Gangaraju R. (2024). Cysteine Leukotriene Receptor Antagonist-Montelukast Effects on Diabetic Retinal Microvascular Endothelial Cells Curtail Autophagy. Investig. Ophthalmol. Vis. Sci..

[19] Prieto-Garcia C., Matkovic V., Mosler T., Li C., Liang J., Oo J.A., Haidle F., Mačinković I., Cabrera-Orefice A., Berkane R. (2024). Pathogenic proteotoxicity of cryptic splicing is alleviated by ubiquitination and ER-phagy. Science.

[20] Azam M., Jastrzebska B. (2025). Mechanisms of Rhodopsin-Related Inherited Retinal Degeneration and Pharmacological TreatmentStrategies. Cells.

[21] Grimes W.N., Berson D.M., Sabnis A., Hoon M., Sinha R., Tian H., Diamond J.S. (2025). Layer-specific anatomical and physiological features of the retina’s neurovascular unit. Curr. Biol..

[22] Qambari H., Hein M., Balaratnasingam C., Yu P., Yu D.Y. (2025). Enabling visualization of GFAP-positive retinal glial cells, neurons and microvasculature in three-dimensions. Exp. Eye Res..

[23] Udom G.J., Oritsemuelebi B., Frazzoli C., Bocca B., Ruggieri F., Orisakwe O.E. (2025). Tyazhelye metally I narusheniya uglevodnogo obmena pri zabolevaniyakh organa zreniya: Sistematicheskii obzor [Heavy metals and derangement in carbohydrate metabolism in eye diseases: A systematic review]. Vestn. Oftalmol..

[24] Yang L., Yao Y., Zheng W., Zheng X., Xie M., Huang L. (2025). Nitric oxide mediates negative feedback on the TXNIP/NLRP3 inflammasome pathway to prevent retinal neurovascular unit dysfunction in early diabetic retinopathy. Free Radic. Biol. Med..

[25] Zhu F., Feng J., Pan Y., Ouyang L., He T., Xing Y. (2025). Mettl3-Mediated N6-Methyladenosine Modification Mitigates Ganglion Cell Loss and Retinal Dysfunction in Retinal Ischemia-Reperfusion Injury by Inhibiting FoxO1-Mediated Autophagy. Investig. Ophthalmol. Vis. Sci..

[26] Pfeiffer R.L., Marc R.E., Jones B.W. (2020). Persistent remodeling and neurodegeneration in late-stage retinal degeneration. Prog. Retin. Eye Res..

[27] Pfeiffer R.L., Marc R.E., Jones B.W. (2020). Müller Cell Metabolic Signatures: Evolutionary Conservation and Disruption in Disease. Trends Endocrinol. Metab..

[28] Pfeiffer R.L., Anderson J.R., Dahal J., Garcia J.C., Yang J.H., Sigulinsky C.L., Rapp K., Emrich D.P., Watt C.B., Johnstun H.A. (2020). A pathoconnectome of early neurodegeneration: Network changes in retinal degeneration. Exp. Eye Res..

[29] Koller A., Lamina C., Brandl C., Zimmermann M.E., Stark K.J., Weissensteiner H., Würzner R., Heid I.M., Kronenberg F. (2023). Systemic Evidence for Mitochondrial Dysfunction in Age-Related Macular Degeneration as Revealed by mtDNA Copy Number Measurements in Peripheral Blood. Int. J. Mol. Sci..

[30] Zhang S.M., Fan B., Li Y.L., Zuo Z.Y., Li G.Y. (2023). Oxidative Stress-Involved Mitophagy of Retinal Pigment Epithelium and Retinal Degenerative Diseases. Cell. Mol. Neurobiol..

[31] Hansman D.S., Du J., Casson R.J., Peet D.J. (2025). Eye on the horizon: The metabolic landscape of the RPE in aging and disease. Prog. Retin. Eye Res..

[32] Deng G., Moran E.P., Cheng R., Matlock G., Zhou K., Moran D., Chen D., Yu Q., Ma J.X. (2017). Therapeutic Effects of a Novel Agonist of Peroxisome Proliferator-Activated Receptor Alpha for the Treatment of Diabetic Retinopathy. Investig. Ophthalmol. Vis. Sci..

[33] Koutsifeli P., Varma U., Daniels L.J., Annandale M., Li X., Neale J.P.H., Hayes S., Weeks K.L., James S., Delbridge L.M.D. (2022). Glycogen-autophagy: Molecular machinery and cellular mechanisms of glycophagy. J. Biol. Chem..

[34] Laqtom N.N., Dong W., Medoh U.N., Cangelosi A.L., Dharamdasani V., Chan S.H., Kunchok T., Lewis C.A., Heinze I., Tang R. (2022). CLN3 is required for the clearance of glycerophosphodiesters from lysosomes. Nature.

[35] Canibano-Fraile R., Harlaar L., Dos Santos C.A., Hoogeveen-Westerveld M., Demmers J.A.A., Snijders T., Lijnzaad P., Verdijk R.M., van der Beek N.A.M.E., van Doorn P.A. (2023). Lysosomal glycogen accumulation in Pompe disease results in disturbed cytoplasmic glycogen metabolism. J. Inherit. Metab..

[36] Mancini M.C., Noland R.C., Collier J.J., Burke S.J., Stadler K., Heden T.D. (2023). Lysosomalglucose sensing and glycophagy in metabolism. Trends Endocrinol. Metab..

[37] Wu L.X., Xu Y.C., Pantopoulos K., Tan X.Y., Wei X.L., Zheng H., Luo Z. (2023). Glycophagy mediated glucose-induced changes of hepatic glycogen metabolism via OGT1-AKT1-FOXO1(Ser238) pathway. J. Nutr. Biochem..

[38] Shatz N., Chohan Y., Klionsky D.J. (2024). ATG14 and STX18: Gatekeepers of lipid droplet degradation and the implications for disease modulation. Autophagy.

[39] Xu Z., Notomi S., Wu G., Fukuda Y., Maehara Y., Fukushima M., Murakami Y., Takahashi M., Izumi Y., Sonoda K.H. (2024). Altered fatty acid distribution in lysosome-associated membraneprotein-2 deficient mice. Biochem. Biophys. Rep..

[40] Zhu L., Guo L., Xu J., Xiang Q., Tan Y., Tian F., Du X., Zhang S., Wen T., Liu L. (2024). Postprandial Triglyceride-Rich Lipoproteins-Induced Lysosomal Dysfunction and Impaired Autophagic Flux Contribute to Inflammation in White Adipocytes. J. Nutr..

[41] Chowdhury O., Bammidi S., Gautam P., Babu V.S., Liu H., Shang P., Xin Y., Mahally E., Nemani M., Koontz V. (2025). Activated mTOR Signaling in the RPE Drives EMT, Autophagy, and Metabolic Disruption, Resulting in AMD-Like Pathology in Mice. Aging Cell.

[42] Jiang F., Ma J., Lei C., Zhang Y., Zhang M. (2025). Age-Related Macular Degeneration: Cellular and Molecular Signaling Mechanisms. Int. J. Mol. Sci..

[43] Lee T.J., Santeford A., Pitts K.M., Ripoll C.V., Terao R., Guo Z., Ozcan M., Kratky D., Christoffersen C., Javaheri A. (2025). Apolipoprotein M attenuates age-related macular degeneration phenotypes via sphingosine-1-phosphate signaling and lysosomal lipid catabolism. Nat. Commun..

[44] Pinelli R., Lazzeri G., Berti C., Biagioni F., Scaffidi E., Ferrucci M., Bumah V.V., Fornai F. (2025). Retinal Autophagy for Sustaining Retinal Integrity as a Proof of Concept for Age-Related Macular Degeneration. Int. J. Mol. Sci..

[45] Wang Z., Anderson D.M.G., Messinger J.D., Curcio C.A., Schey K.L. (2025). Glycolipids implicated as mediators of clinically visible retinal pigment epithelial migration in age-related macular degeneration. Proc. Natl. Acad. Sci. USA.

[46] Li C.M., Clark M.E., Rudolf M., Curcio C.A. (2007). Distribution and composition of esterified and unesterified cholesterol in extra-macular drusen. Exp. Eye Res..

[47] Golestaneh N., Chu Y., Xiao Y.Y., Stoleru G.L., Theos A.C. (2017). Dysfunctional autophagy in RPE, a contributing factor in age-related macular degeneration. Cell Death Dis..

[48] Giorgianni F., Beranova-Giorgianni S. (2022). Oxidized low-density lipoprotein causes ribosome reduction and inhibition of protein synthesis in retinal pigment epithelial cells. Biochem. Biophys. Rep..

[49] Luo Z., Saha A.K., Xiang X., Ruderman N.B. (2005). AMPK, the metabolic syndrome and cancer. Trends Pharmacol. Sci..

[50] Laplante M., Sabatini D.M. (2012). mTOR signaling in growth control and disease. Cell.

[51] Wu C.C., Chou P., Jacinto E., Da Silva Xavier G. (2012). The target of rapamycin: Structure and functions. Protein Kinases.

[52] Yuan Y., Pan B., Sun H., Chen G., Su B., Huang Y. (2015). Characterization of Sin1 isoforms reveals an mTOR-dependent and independent function of Sin1gamma. PLoS ONE.

[53] Szwed A., Kim E., Jacinto E. (2021). Regulation and metabolic functions of mTORC1 and mTORC2. Physiol. Rev..

[54] Movahhed P., Saberiyan M., Safi A., Arshadi Z., Kazerouni F., Teimori H. (2022). The impact of DAPK1 and mTORC1 signaling association on autophagy in cancer. Mol. Biol. Rep..

[55] Stanciu S.M., Jinga M., Miricescu D., Stefani C., Nica R.I., Stanescu-Spinu I.I., Vacaroiu I.A., Greabu M., Nica S. (2024). mTOR Dysregulation, Insulin Resistance, and Hypertension. Biomedicines.

[56] Noda T. (2017). Regulation of Autophagy through TORC1 and mTORC1. Biomolecules.

[57] Bodineau C., Tomé M., Murdoch P.D.S., Durán R.V. (2022). Glutamine, MTOR and autophagy: A multiconnection relationship. Autophagy.

[58] Hertel A., Alves L.M., Dutz H., Tascher G., Bonn F., Kaulich M., Dikic I., Eimer S., Steinberg F., Bremm A. (2022). USP32-regulated LAMTOR1 ubiquitination impacts mTORC1 activation and autophagy induction. Cell Rep..

[59] Huang P., Dong R.Y., Wang P., Xu M., Sun X., Dong X.P. (2024). MCOLN/TRPML channels in the regulation of MTORC1 and autophagy. Autophagy.

[60] Jacinto E., Loewith R., Schmidt A., Lin S., Ruegg M.A., Hall A., Hall M.N. (2004). Mammalian TOR complex 2 controls the actin cytoskeleton and is rapamycin insensitive. Nat. Cell Biol..

[61] Merhi A., Delree P., Marini A.M. (2017). The metabolic waste ammonium regulates mTORC2 and mTORC1 signaling. Sci. Rep..

[62] Hatano T., Morigasaki S., Tatebe H., Ikeda K., Shiozaki K. (2015). Fission yeast Ryh1 GTPase activates TOR Complex 2 in response to glucose. Cell Cycle.

[63] Kazyken D., Magnuson B., Bodur C., Acosta-Jaquez H.A., Zhang D., Tong X., Barnes T.M., Steinl G.K., Patterson N.E., Altheim C.H. (2019). AMPK directly activates mTORC2 to promote cell survival during acute energetic stress. Sci. Signal..

[64] Heimbucher T., Qi W., Baumeister R. (2020). TORC2-SGK-1 signaling integrates external signals to regulate autophagic turnover of mitochondria via mtROS. Autophagy.

[65] Xu J., Wang G., Hou Y., Sun K., Zheng Z., Guo Z., Hou L., Zhang X., Ruan Z., Ye Y. (2025). RICTOR-mediated activation of AKT/mTOR signaling and autophagy inhibition promote osteoarthritis. Int. Immunopharmacol..

[66] Zhang S., Li G., He L., Wang F., Gao M., Dai T., Su Y., Li L., Cao Y., Zheng M. (2025). Sphingosine kinase 2 deficiency impairs VLDL secretion by inhibiting mTORC2 phosphorylation and activating chaperone-mediated autophagy. Cell Death Differ..

[67] Feng D., Gui Z., Xu Z., Zhang J., Ni B., Wang Z., Liu J., Fei S., Chen H., Sun L. (2024). Rictor/mTORC2 signalling contributes to renal vascular endothelial-to-mesenchymal transition and renal allograft interstitial fibrosis by regulating BNIP3-mediated mitophagy. Clin. Transl. Med..

[68] Iskandar K., Foo J., Liew A.Q.X., Zhu H., Raman D., Hirpara J.L., Leong Y.Y., Babak M.V., Kirsanova A.A., Armand A.S. (2024). A novel MTORC2-AKT-ROS axis triggers mitofission and mitophagy-associated execution of colorectal cancer cells upon drug-induced activation of mutant KRAS. Autophagy.

[69] Park Y.J., Cho M.Y., Kim I.Y., Lee D.M., Yoon G., Choi K.S. (2025). mTORC2/Akt axis promotes proteotoxic stress and mitochondrial Ca^2+^ overload during celastrol-induced paraptosis. Biochem. Biophys. Res. Commun..

[70] Ferese R., Lenzi P., Fulceri F., Biagioni F., Fabrizi C., Gambardella S., Familiari P., Frati A., Limanaqi F., Fornai F. (2020). Quantitative Ultrastructural Morphometry and Gene Expression of mTOR-Related Mitochondriogenesis within Glioblastoma Cells. Int. J. Mol. Sci..

[71] Lenzi P., Ferese R., Biagioni F., Fulceri F., Busceti C.L., Falleni A., Gambardella S., Frati A., Fornai F. (2021). Rapamycin Ameliorates Defects in Mitochondrial Fission and Mitophagy in Glioblastoma Cells. Int. J. Mol. Sci..

[72] Haythorne E., Lloyd M., Walsby-Tickle J., Tarasov A.I., Sandbrink J., Portillo I., Exposito R.T., Sachse G., Cyranka M., Rohm M. (2022). Altered glycolysis triggers impaired mitochondrial metabolism and mTORC1 activation in diabetic β-cells. Nat. Commun..

[73] Bhandari S., Kim Y.I., Nam I.K., Hong K., Jo Y., Yoo K.W., Liao W., Lim J.Y., Kim S.J., Um J.Y. (2023). Loss of pex5 sensitizes zebrafish to fasting due to deregulated mitochondria, mTOR, and autophagy. Cell. Mol. Life Sci..

[74] Kalenta H., Kilroe S.P., Romsdahl T.B., Marchant E.D., Maroto R., Linares J.J., Russell W.K., Rasmussen B.B. (2025). Constitutively active mTORC1 signaling modifies the skeletal muscle metabolome and lipidome response to exercise. J. Appl. Physiol..

[75] Kim H., Massett M.P. (2024). Beneficial effects of rapamycin on endothelial function in systemic lupus erythematosus. Front. Physiol..

[76] Chung C.Y., Singh K., Sheshadri P., Valdebenito G.E., Chacko A.R., Costa Besada M.A., Liang X.F., Kabir L., Pitceathly R.D.S., Szabadkai G. (2025). Inhibition of the PI3K-AKT-MTORC1 axis reduces the burden of the m.3243A>G mtDNA mutation by promoting mitophagy and improving mitochondrial function. Autophagy.

[77] de la Cruz López K.G., Toledo Guzmán M.E., Sánchez E.O., García Carrancá A. (2019). mTORC1 as a Regulator of Mitochondrial Functions and a Therapeutic Target in Cancer. Front. Oncol..

[78] Li Y., Wu P., Dai J., Zhang T., Bihl J., Wang C., Liu Y., Shi H. (2020). Inhibition of mTOR Alleviates Early Brain Injury After Subarachnoid Hemorrhage Via Relieving Excessive Mitochondrial Fission. Cell. Mol. Neurobiol..

[79] Hei C., Zhou Y., Zhang C., Gao F., Cao M., Yuan S., Qin Y., Li P.A., Yang X. (2023). Rapamycin ameliorates brain damage and maintains mitochondrial dynamic balance in diabetic rats subjected to middle cerebral artery occlusion. Metab. Brain Dis..

[80] Wu Y., Zhou K., Liu B., Xu J., Lei L., Hu J., Cheng X., Zhong F., Wang S. (2023). Glial Activation, Mitochondrial Imbalance, and Akt/mTOR Signaling May Be Potential Mechanisms of Cognitive Impairment in Heart Failure Mice. Neurotox. Res..

[81] Zhong W.J., Zhang J., Duan J.X., Zhang C.Y., Ma S.C., Li Y.S., Yang N.S., Yang H.H., Xiong J.B., Guan C.X. (2023). TREM-1 triggers necroptosis of macrophages through mTOR-dependent mitochondrial fission during acute lung injury. J. Transl. Med..

[82] Prajapati S.K., Ahmed S., Rai V., Gupta S.C., Krishnamurthy S. (2024). Suvorexant improves mitochondrial dynamics with the regulation of orexinergic and mTOR activation in rats exhibiting PTSD-like symptoms. J. Affect. Disord..

[83] Bockaert J., Marin P. (2015). mTOR in Brain Physiology and Pathologies. Physiol. Rev..

[84] Powers T. (2022). The origin story of rapamycin: Systemic bias in biomedical research and cold war politics. Mol. Biol. Cell.

[85] Seglen P.O., Gordon P.B. (1982). 3-Methyladenine: Specific inhibitor of autophagic/lysosomal protein degradation in isolated rat hepatocytes. Proc. Natl. Acad. Sci. USA.

[86] Petiot A., Ogier-Denis E., Blommaart E.F., Meijer A.J., Codogno P. (2000). Distinct classes of phosphatidylinositol 3’-kinases are involved in signaling pathways that control macroautophagy in HT-29 cells. J. Biol. Chem..

[87] Klionsky D.J., Abdel-Aziz A.K., Abdelfatah S., Abdellatif M., Abdoli A., Abel S., Abeliovich H., Abildgaard M.H., Abudu Y.P., Acevedo-Arozena A. (2021). Guidelines for the use and interpretation of assays for monitoring autophagy (4th edition). Autophagy.

[88] Nascimbeni A.C., Codogno P., Morel E. (2017). Phosphatidylinositol-3-phosphate in the regulation of autophagy membrane dynamics. FEBS J..

[89] Po W.W., Thein W., Khin P.P., Khing T.M., Han K.W.W., Park C.H., Sohn U.D. (2020). Fluoxetine Simultaneously Induces Both Apoptosis and Autophagy in Human Gastric Adenocarcinoma Cells. Biomol. Ther..

[90] Fabrizi C., Somma F., Pompili E., Biagioni F., Lenzi P., Fornai F., Fumagalli L. (2012). Role of autophagy inhibitors and inducers in modulating the toxicity of trimethyltin in neuronal cell cultures. J. Neural. Transm..

[91] Lenzi P., Lazzeri G., Ferrucci M., Busceti C.L., Puglisi-Allegra S., Fornai F. (2024). In situ stoichiometry amounts of p62 and poly-ubiquitin exceed the increase of alpha-synuclein during degeneration of catecholamine cells induced by autophagy inhibition in vitro. J. Neural. Transm..

[92] Prasad S., Aggarwal B.B., Benzie I.F.F., Wachtel-Galor S. (2011). Turmeric, the Golden Spice: From Traditional Medicine to Modern Medicine. Herbal Medicine: Biomolecular and Clinical Aspects.

[93] Prasad S., Gupta S.C., Tyagi A.K., Aggarwal B.B. (2014). Curcumin, a component of golden spice: From bedside to bench and back. Biotechnol. Adv..

[94] Ghalandarlaki N., Alizadeh A.M., Ashkani-Esfahani S. (2014). Nanotechnology-applied curcumin for different diseases therapy. Biomed. Res. Int..

[95] Hewlings S., Kalman D. (2017). Curcumin: A Review of Its’ Effects on Human Health. Foods.

[96] Ayati Z., Ramezani M., Amiri M.S., Moghadam A.T., Rahimi H., Abdollahzade A., Sahebkar A., Emami S.A. (2019). Ethnobotany, phytochemistry and traditional uses of *Curcuma* spp. and pharmacological profile of two important species (*C. longa* and *C. zedoaria*): A review. Curr. Pharmaceut. Des..

[97] Askarizadeh A., Barreto G.E., Henney N.C., Majeed M., Sahebkar A. (2020). Neuroprotection by curcumin: A review on brain delivery strategies. Int. J. Pharm..

[98] Akaberi M., Sahebkar A., Emami S.A. (2021). Turmeric and Curcumin: From Traditional to Modern Medicine. Adv. Exp. Med. Biol..

[99] Nunes Y.C., Mendes N.M., Pereira de Lima E., Chehadi A.C., Lamas C.B., Haber J.F.S., Dos Santos Bueno M., Araújo A.C., Catharin V.C.S., Detregiachi C.R.P. (2024). Curcumin: A Golden Approach to Healthy Aging: A Systematic Review of the Evidence. Nutrients.

[100] Khayatan D., Razavi S.M., Arab Z.N., Nasoori H., Fouladi A., Pasha A.V.K., Butler A.E., Karav S., Momtaz S., Abdolghaffari A.H. (2025). Targeting mTOR with curcumin: Therapeutic implications for complex diseases. Inflammopharmacology.

[101] Memarzia A., Khazdair M.R., Behrouz S., Gholamnezhad Z., Jafarnezhad M., Saadat S., Boskabady M.H. (2021). Experimental and clinical reports on anti-inflammatory, antioxidant, and immunomodulatory effects of Curcuma longa and curcumin, an updated and comprehensive review. Biofactors.

[102] Ryskalin L., Puglisi-Allegra S., Lazzeri G., Biagioni F., Busceti C.L., Balestrini L., Fornasiero A., Leone S., Pompili E., Ferrucci M. (2021). Neuroprotective Effects of Curcumin in Methamphetamine-Induced Toxicity. Molecules.

[103] Yuan R., Li Y., Han S., Chen X., Chen J., He J., Gao H., Yang Y., Yang S., Yang Y. (2022). Fe-Curcumin Nanozyme-Mediated Reactive Oxygen Species Scavenging and Anti-Inflammation for Acute Lung Injury. ACS Cent. Sci..

[104] Asif H.M., Zafar F., Ahmad K., Iqbal A., Shaheen G., Ansari K.A., Rana S., Zahid R., Ghaffar S. (2023). Synthesis, characterization and evaluation of anti-arthritic and anti-inflammatory potential of curcumin loaded chitosan nanoparticles. Sci. Rep..

[105] Borges G.A., Elias S.T., Amorim B., Lima C.L., Coletta R.D., Castilho R.M., Squarize C.H., Guerra E.N.S. (2020). Curcumin Downregulates the PI3K–AKT–mTOR Pathway and Inhibits Growth and Progression in Head and Neck Cancer Cells. Phytother. Res..

[106] Yang L., Shi J., Wang X., Zhang R. (2022). Curcumin alleviates D-galactose-induced cardiomyocyte senescence by promoting autophagy via the SIRT1/AMPK/mTOR pathway. Evid. Based Complement. Altern. Med..

[107] Warias P., Plewa P., Poniewierska-Baran A. (2024). Resveratrol, piceatannol, curcumin, and Quercetin as therapeutic targets in gastric Cancer—Mechanisms and clinical implications for natural products. Molecules.

[108] Wang S., Wang X., Zheng X., Jiang H., Liu L., Ma N., Dong X. (2025). NGR-modified curcumin nanovesicles reverse immunotherapy resistance in triple-negative breast cancer via TLR9 and mTOR pathway modulation. Cell Biol. Toxicol..

[109] Poot M., Zhang Y.Z., Kramer J.A., Wells K.S., Jones L.J., Hanzel D.K., Lugade A.G., Singer V.L., Haugland R.P. (1996). Analysis of mitochondrial morphology and function with novel fixable fluorescent stains. J. Histochem. Cytochem. Off. J. Histochem. Soc..

[110] Presley A.D., Fuller K.M., Arriaga E.A. (2003). MitoTracker Green labeling of mitochondrial proteins and their subsequent analysis by capillary electrophoresis with laser-induced fluorescence detection. J. Chromatogr. B Anal. Technol. Biomed. Life Sci..

[111] Gautam N., Sankaran S., Yason J.A., Tan K.S.W., Gascoigne N.R.J. (2018). A high content imaging flow cytometry approach to study mitochondria in T cells: MitoTracker Green FM dye concentration optimization. Methods.

[112] Clutton G., Mollan K., Hudgens M., Goonetilleke N. (2019). A Reproducible, Objective Method Using MitoTracker^®^ Fluorescent Dyes to Assess Mitochondrial Mass in T Cells by Flow Cytometry. Cytom. A.

[113] Chazotte B. (2011). Labeling mitochondria with MitoTracker dyes. Cold Spring Harb. Protoc..

[114] Perry S.W., Norman J.P., Barbieri J., Brown E.B., Gelbard H.A. (2011). Mitochondrial membrane potential probes and the proton gradient: A practical usage guide. Biotechniques.

[115] Zhitomirsky B., Farber H., Assaraf Y.G. (2018). LysoTracker and MitoTracker Red are transport substrates of P-glycoprotein: Implications for anticancer drug design evading multidrug resistance. J. Cell. Mol. Med..

[116] Martins W.K., Santos N.F., Rocha C.S., Bacellar I.O.L., Tsubone T.M., Viotto A.C., Matsukuma A.Y., Abrantes A.B.P., Siani P., Dias L.G. (2019). Parallel damage in mitochondria and lysosomes is an efficient way to photoinduce cell death. Autophagy.

[117] Lenzi P., Marongiu R., Falleni A., Gelmetti V., Busceti C.L., Michiorri S., Valente E.M., Fornai F. (2012). A subcellular analysis of genetic modulation of PINK1 on mitochondrial alterations, autophagy and cell death. Arch. Ital. Biol..

[118] Moore D., West A.B., Dawson V.L., Dawson T.M. (2005). Molecular pathophysiology of Parkinson’s disease. Annu. Rev. Neurosci..

[119] Dawson T.M. (2006). Parkin and defective ubiquitination in Parkinson’s disease. J. Neural. Transm. Suppl..

[120] Pirooznia S.K., Yuan C., Khan M.R., Karuppagounder S.S., Wang L., Xiong Y., Kang S.U., Lee Y., Dawson V.L., Dawson T.M. (2020). PARIS induced defects in mitochondrial biogenesis drive dopamine neuron loss under conditions of parkin or PINK1 deficiency. Mol. Neurodegener..

[121] Lenzi P., Biagioni F., Busceti C.L., Lazzeri G., Polzella M., Frati A., Ferrucci M., Fornai F. (2022). Alterations of Mitochondrial Structure in Methamphetamine Toxicity. Int. J. Mol. Sci..

[122] Narendra D.P., Youle R.J. (2024). The role of PINK1-Parkin in mitochondrial quality control. Nat. Cell Biol..

[123] Watzlawik J.O., Fiesel F.C., Fiorino G., Bustillos B.A., Baninameh Z., Markham B.N., Hou X., Hayes C.S., Bredenberg J.M., Kurchaba N.W. (2024). Basal activity of PINK1 and PRKN in cell models and rodent brain. Autophagy.

[124] Basak B., Holzbaur E.L.F. (2025). Mitophagy in Neurons: Mechanisms Regulating Mitochondrial Turnover and Neuronal Homeostasis. J. Mol. Biol..

[125] Yang H., Wang Y., Liu S., Zhang S., Chen Y., Ding J., Chen S., Zhu F., Xia B., Luo P. (2025). Polysaccharide alleviates neurodegeneration and behavioral deficit by enhancing mitochondrial autophagy in chronic methamphetamine mice. Neurotoxicology.

[126] Hyttinen J., Blasiak J., Tavi P., Kaarniranta K. (2021). Therapeutic potential of PGC-1α in age-related macular degeneration (AMD)—The involvement of mitochondrial quality control, autophagy, and antioxidant response. Expert. Opin. Ther. Targets.

[127] Lewis Luján L.M., McCarty M.F., Di Nicolantonio J.J., Gálvez Ruiz J.C., Rosas-Burgos E.C., Plascencia-Jatomea M., Iloki Assanga S.B. (2022). Nutraceuticals/Drugs Promoting Mitophagy and Mitochondrial Biogenesis May Combat the Mitochondrial Dysfunction Driving Progression of Dry Age-Related Macular Degeneration. Nutrients.

[128] Aoki H., Takada Y., Kondo S., Sawaya R., Aggarwal B.B., Kondo Y. (2007). Evidence that curcumin suppresses the growth of malignantgliomas in vitro and in vivo through induction of autophagy: Role ofAkt and extracellular signal-regulated kinase signaling pathways. Mol. Pharmacol..

[129] Sekiguchi A., Kanno H., Ozawa H., Yamaya S., Itoi E. (2012). Rapamycin promotes autophagy and reduces neural tissue damage and locomotor impairment after spinal cord injury in mice. J. Neurotrauma.

[130] Viscomi M.T., D’Amelio M., Cavallucci V., Latini L., Bisicchia E., Nazio F., Fanelli F., Maccarrone M., Moreno S., Cecconi F. (2012). Stimulation of autophagy by rapamycin protects neurons from remote degeneration after acute focal brain damage. Autophagy.

[131] Zhuang W., Long L., Zheng B., Ji W., Yang N., Zhang Q., Liang Z. (2012). Curcumin promotes differentiation of glioma-initiating cells byinducing autophagy. Cancer Sci..

[132] Meng Y., Yong Y., Yang G., Ding H., Fan Z., Tang Y., Luo J., Ke Z.J. (2013). Autophagy alleviates neurodegeneration caused by mild impairment of oxidative metabolism. J. Neurochem..

[133] Heras-Sandoval D., Pérez-Rojas J.M., Hernández-Damián J., Pedraza-Chaverri J. (2014). The role of PI3K/AKT/mTOR pathway in the modulation of autophagy and the clearance of protein aggregates in neurodegeneration. Cell. Signal..

[134] Zhao G., Han X., Zheng S., Li Z., Sha Y., Ni J., Ni J., Sun Z., Qiao S., Song Z. (2016). Curcumin induces autophagy, inhibits proliferation and invasion bydownregulating AKT/mTOR signaling pathway in human melanomacells. Oncol. Rep..

[135] Li W., Zhou Y., Yang J., Li H., Zhang H., Zheng P. (2017). Curcumin induces apoptotic cell death and protective autophagy in humangastric cancer cells. Oncol. Rep..

[136] Shakeri A., Cicero A.F.G., Panahi Y., Mohajeri M., Sahebkar A. (2019). Curcumin: A naturally occurring autophagy modulator. J. Cell. Physiol..

[137] Singh A.K., Singh S., Tripathi V.K., Bissoyi A., Garg G., Rizvi S.I. (2019). Rapamycin Confers Neuroprotection Against Aging-Induced Oxidative Stress, Mitochondrial Dysfunction, and Neurodegeneration in Old Rats Through Activation of Autophagy. Rejuvenation Res..

[138] Islam M.R., Rauf A., Akter S., Akter H., Al-Imran M.I.K., Fakir M.N.H., Thufa G.K., Islam M.T., Hemeg H.A., Abdulmonem W.A. (2025). Neuroprotective Potential of Curcumin in Neurodegenerative Diseases: Clinical Insights into Cellular and Molecular Signaling Pathways. J. Biochem. Mol. Toxicol..

[139] Bové J., Martínez-Vicente M., Vila M. (2011). Fighting neurodegeneration with rapamycin: Mechanistic insights. Nat. Rev. Neurosci..

[140] Senapati P.K., Mahapatra K.K., Singh A., Bhutia S.K. (2025). mTOR inhibitors in targeting autophagy and autophagy-associated signaling for cancer cell death and therapy. Biochim. Biophys. Acta Rev. Cancer.

[141] Yao J., Jia L., Khan N., Lin C., Mitter S.K., Boulton M.E., Dunaief J.L., Klionsky D.J., Guan J.L., Thompson D.A. (2015). Deletion of autophagy inducer RB1CC1 results in degeneration of the retinal pigment epithelium. Autophagy.

[142] Sun C.K., Kao C.T., Wei M.L., Chia S.H., Kärtner F.X., Ivanov A., Liao Y.H. (2019). Slide-free imaging of hematoxylin-eosin stained whole-mount tissues using combined third-harmonic generation and three-photon fluorescence microscopy. J. Biophotonics.

[143] Borah B.J., Tseng Y.C., Wang K.C., Wang H.C., Huang H.Y., Chang K., Lin J.R., Liao Y.H., Sun C.K. (2023). Rapid digital pathology of H&E-stained fresh human brain specimens as an alternative to frozen biopsy. Commun. Med..

[144] Georgiadis A., Tschernutter M., Bainbridge J.W., Balaggan K.S., Mowat F., West E.L., Munro P.M., Thrasher A.J., Matter K., Balda M.S. (2010). The tight junction associated signalling proteins ZO-1 and ZONAB regulate retinal pigment epithelium homeostasis in mice. PLoS ONE.

[145] Shirasawa M., Sonoda S., Terasaki H., Arimura N., Otsuka H., Yamashita T., Uchino E., Hisatomi T., Ishibashi T., Sakamoto T. (2013). TNF-α disrupts morphologic and functional barrier properties of polarized retinal pigment epithelium. Exp. Eye Res..

[146] Chowers G., Cohen M., Marks-Ohana D., Stika S., Eijzenberg A., Banin E., Obolensky A. (2017). Course of Sodium Iodate-Induced Retinal Degeneration in Albino and Pigmented Mice. Investig. Ophthalmol. Vis. Sci..

[147] Maugeri G., D’Amico A.G., Rasà D.M., La Cognata V., Saccone S., Federico C., Cavallaro S., D’Agata V. (2017). Caffeine Prevents Blood Retinal Barrier Damage in a Model, In Vitro, of Diabetic Macular Edema. J. Cell. Biochem..

[148] Obert E., Strauss R., Brandon C., Grek C., Ghatnekar G., Gourdie R., Rohrer B. (2017). Targeting the tight junction protein, zonula occludens-1, with the connexin43 mimetic peptide, αCT1, reduces VEGF-dependent RPE pathophysiology. J. Mol. Med..

[149] Ozkaya E.K., Anderson G., Dhillon B., Bagnaninchi P.O. (2019). Blue-light induced breakdown of barrier function on human retinal epithelial cells is mediated by PKC-ζ over-activation and oxidative stress. Exp. Eye Res..

[150] Matsubara J.A., Tian Y., Cui J.Z., Zeglinski M.R., Hiroyasu S., Turner C.T., Granville D.J. (2020). Retinal Distribution and Extracellular Activity of Granzyme B: A Serine Protease That Degrades Retinal Pigment Epithelial Tight Junctions and Extracellular Matrix Proteins. Front. Immunol..

[151] Paterson C., Cannon J., Vargis E. (2023). The impact of early RPE cell junction loss on VEGF, Ang-2, and TIMP secretion in vitro. Mol. Vis..

[152] Ye Y.T., Niu Y.L., Zhou Z.Y., Sun Y., Chang T.F., Jing Y.T., Bai Q., Chu Z.J. (2024). Carbonic anhydrase inhibitor alleviates retinal barrier toxicity in paclitaxel-induced retinopathy and macular edema by inhibiting CAXIV. Int. Ophthalmol..

[153] Kandezi N., Mohammadi M., Ghaffari M., Gholami M., Motaghinejad M., Safari S. (2020). Novel Insight to Neuroprotective Potential of Curcumin: A Mechanistic Review of Possible Involvement of Mitochondrial Biogenesis and PI3/Akt/GSK3 or PI3/Akt/CREB/BDNF Signaling Pathways. Int. J. Mol. Cell. Med..

[154] Hamidie R.D.R., Shibaguchi T., Yamada T., Koma R., Ishizawa R., Saito Y., Hosoi T., Masuda K. (2021). Curcumin induces mitochondrial biogenesis by increasing cyclic AMP levels via phosphodiesterase 4A inhibition in skeletal muscle. Br. J. Nutr..

[155] Rahman M.A., Akter S., Dorotea D., Mazumder A., Uddin M.N., Hannan M.A., Hossen M.J., Ahmed M.S., Kim W., Kim B. (2022). Renoprotective potentials of small molecule natural products targeting mitochondrial dysfunction. Front. Pharmacol..

[156] Zheng Q., Liu H., Zhang H., Han Y., Yuan J., Wang T., Gao Y., Li Z. (2023). Ameliorating Mitochondrial Dysfunction of Neurons by Biomimetic Targeting Nanoparticles Mediated Mitochondrial Biogenesis to Boost the Therapy of Parkinson’s Disease. Adv. Sci..

[157] Hou D., Liao H., Hao S., Liu R., Huang H., Duan C. (2024). Curcumin simultaneously improves mitochondrial dynamics and myocardial cell bioenergy after sepsis via the SIRT1-DRP1/PGC-1α pathway. Heliyon.

[158] Palabiyik A.A., Palabiyik E. (2025). Pharmacological approaches to enhance mitochondrial biogenesis: Focus on PGC-1A, AMPK, and SIRT1 in cellular health. Mol. Biol. Rep..

[159] Perez-Pinzon M.A., Stetler R.A., Fiskum G. (2012). Novel mitochondrial targets for neuroprotection. J. Cereb. Blood Flow Metab..

[160] Palikaras K., Lionaki E., Tavernarakis N. (2015). Coupling mitogenesis and mitophagy for longevity. Autophagy.

[161] Palikaras K., Lionaki E., Tavernarakis N. (2015). Balancing mitochondrial biogenesis and mitophagy to maintain energy metabolism homeostasis. Cell Death Differ..

[162] Palikaras K., Lionaki E., Tavernarakis N. (2015). Mitophagy: In sickness and in health. Mol. Cell. Oncol..

[163] Palikaras K., Daskalaki I., Markaki M., Tavernarakis N. (2017). Mitophagy and age-related pathologies: Development of new therapeutics by targeting mitochondrial turnover. Pharm. Ther..

[164] Civiletto G., Dogan S.A., Cerutti R., Fagiolari G., Moggio M., Lamperti C., Benincá C., Viscomi C., Zeviani M. (2018). Rapamycin rescues mitochondrial myopathy via coordinated activation of autophagy and lysosomal biogenesis. EMBO Mol. Med..

[165] Markaki M., Palikaras K., Tavernarakis N. (2018). Novel Insights into the Anti-aging Role of Mitophagy. Int. Rev. Cell Mol. Biol..

[166] Yerra V.G., Kalvala A.K., Sherkhane B., Areti A., Kumar A. (2018). Adenosine monophosphate- activated protein kinase modulation by berberine attenuates mitochondrial deficits and redox imbalance in experimental diabetic neuropathy. Neuropharmacology.

[167] Dusabimana T., Kim S.R., Kim H.J., Park S.W., Kim H. (2019). Nobiletin ameliorates hepatic ischemia and reperfusion injury through the activation of SIRT-1/FOXO3a-mediated autophagy and mitochondrial biogenesis. Exp. Mol. Med..

[168] Huwatibieke B., Yin W., Liu L., Jin Y., Xiang X., Han J., Zhang W., Li Y. (2021). Mammalian Target of Rapamycin Signaling Pathway Regulates Mitochondrial Quality Control of Brown Adipocytes in Mice. Front. Physiol..

[169] Infante B., Bellanti F., Correale M., Pontrelli P., Franzin R., Leo S., Calvaruso M., Mercuri S., Netti G.S., Ranieri E. (2021). mTOR inhibition improves mitochondria function/biogenesis and delays cardiovascular aging in kidney transplant recipients with chronic graft dysfunction. Aging.

[170] Shen W., Jia N., Miao J., Chen S., Zhou S., Meng P., Zhou X., Tang L., Zhou L. (2021). Penicilliumin B Protects against Cisplatin-Induced Renal Tubular Cell Apoptosis through Activation of AMPK-Induced Autophagy and Mitochondrial Biogenesis. Kidney Dis..

[171] Khan I., Preeti K., Kumar R., Kumar Khatri D., Bala Singh S. (2023). Piceatannol promotes neuroprotection by inducing mitophagy and mitobiogenesis in the experimental mdiabetic peripheral neuropathy and hyperglycemia-induced neurotoxicity. Int. Immunopharmacol..

[172] Khidr H.Y., Hassan N.F., Abdelrahman S.S., El-Ansary M.R., El-Yamany M.F., Rabie M.A. (2023). Formoterol attenuated mitochondrial dysfunction in rotenone-induced Parkinson’s disease in a rat model: Role of PINK-1/PARKIN and PI3K/Akt/CREB/BDNF/TrKB axis. Int. Immunopharmacol..

[173] Jhuo C.F., Chen C.J., Tzen J.T.C., Chen W.Y. (2024). Teaghrelin protected dopaminergic neurons in MPTP-induced Parkinson’s disease animal model by promoting PINK1/Parkin-mediated mitophagy and AMPK/SIRT1/PGC1-α-mediated mitochondrial biogenesis. Environ. Toxicol..

[174] Wu A.G., Yong Y.Y., He C.L., Li Y.P., Zhou X.Y., Yu L., Chen Q., Lan C., Liu J., Yu C.L. (2024). Novel 18-norspirostane steroidal saponins: Extending lifespan and mitigating neurodegeneration through promotion of mitophagy and mitochondrial biogenesis in Caenorhabditis elegans. Mech. Ageing Dev..

[175] Varga K., Sikur N., Paszternák A., Friesenhahn A.L., Zymela F.E., Bagaméry F., Tábi T., Wölfl S. (2025). Resveratrol restores insulin signaling and balances mitochondrial biogenesis and autophagy in streptozotocin-induced neurodegeneration in vitro. Eur. J. Pharm. Sci..

[176] Xi H., Shan W., Li M., Wang Z., Li Y. (2025). Trehalose attenuates testicular aging by activating autophagy and improving mitochondrial quality. Andrology.

[177] Fimia G.M., Stoykova A., Romagnoli A., Giunta L., Di Bartolomeo S., Nardacci R., Corazzari M., Fuoco C., Ucar A., Schwartz P. (2007). Ambra1 regulates autophagy and development of the nervous system. Nature.

[178] Sepe S., Nardacci R., Fanelli F., Rosso P., Bernardi C., Cecconi F., Mastroberardino P.G., Piacentini M., Moreno S. (2014). Expression of Ambra1 in mouse brain during physiological and Alzheimer type aging. Neurobiol. Aging.

[179] Bell K., Rosignol I., Sierra-Filardi E., Rodriguez-Muela N., Schmelter C., Cecconi F., Grus F., Boya P. (2020). Age related retinal Ganglion cell susceptibility in context of autophagy deficiency. Cell Death Discov..

[180] Zhou J., Zhao Y., Li Z., Zhu M., Wang Z., Li Y., Xu T., Feng D., Zhang S., Tang F. (2020). miR-103a-3p regulates mitophagy in Parkinson’s disease through Parkin/Ambra1 signaling. Pharmacol. Res..

[181] Di Rienzo M., Romagnoli A., Refolo G., Vescovo T., Ciccosanti F., Zuchegna C., Lozzi F., Occhigrossi L., Piacentini M., Fimia G.M. (2024). Role of AMBRA1 in mitophagy regulation: Emerging evidence in aging-related diseases. Autophagy.

[182] Palikaras K., Tavernarakis N. (2014). Mitochondrial homeostasis: The interplay between mitophagy and mitochondrial biogenesis. Exp. Gerontol..

[183] Liu L., Li Y., Chen G., Chen Q. (2023). Crosstalk between mitochondrial biogenesis and mitophagy to maintain mitochondrial homeostasis. J. Biomed. Sci..

[184] Xia C., Zhang J., Chen H., Jiang W., Zhou S., Zheng H., Sun W. (2025). Kaempferol improves mitochondrial homeostasis via mitochondrial dynamics and mitophagy in diabetic kidney disease. Int. Immunopharmacol..

[185] Fisher R.P., Topper J.N., Clayton D.A. (1987). Promoter selection in human mitochondria involves binding of a transcription factor to orientation-independent upstream regulatory elements. Cell.

[186] Dairaghi D.J., Shadel G.S., Clayton D.A. (1995). Addition of a 29 residue carboxyl-terminal tail converts a simple HMG box-containing protein into a transcriptional activator. J. Mol. Biol..

[187] Bonawitz N.D., Clayton D.A., Shadel G.S. (2006). Initiation and beyond: Multiple functions of the human mitochondrial transcription machinery. Mol. Cell.

[188] Cunningham J.T., Rodgers J.T., Arlow D.H., Vazquez F., Mootha V.K., Puigserver P. (2007). mTOR controls mitochondrial oxidative function through a YY1-PGC-1alpha transcriptional complex. Nature.

[189] Scarpulla R.C. (2011). Metabolic control of mitochondrial biogenesis through the PGC-1 family regulatory network. Biochim. Biophys. Acta.

[190] Blättler S.M., Verdeguer F., Liesa M., Cunningham J.T., Vogel R.O., Chim H., Liu H., Romanino K., Shirihai O.S., Vazquez F. (2012). Defective mitochondrial morphology and bioenergetic function in mice lacking the transcription factor Yin Yang 1 in skeletal muscle. Mol. Cell. Biol..

[191] Jager S., Handschin C., St-Pierre J., Spiegelman B.M. (2007). AMP-activated protein kinase (AMPK) action in skeletal muscle via direct phosphorylation of PGC-1alpha. Proc. Natl. Acad. Sci. USA.

[192] Canto C., Gerhart-Hines Z., Feige J.N., Lagouge M., Noriega L., Milne J.C., Elliott P.J., Puigserver P., Auwerx J. (2009). AMPK regulates energy expenditure by modulating NAD^+^ metabolism and SIRT1 activity. Nature.

[193] Steinberg G.R., Kemp B.E. (2009). AMPK in Health and Disease. Physiol. Rev..

[194] Kaarniranta K., Uusitalo H., Blasiak J., Felszeghy S., Kannan R., Kauppinen A., Salminen A., Sinha D., Ferrington D. (2020). Mechanisms of mitochondrial dysfunction and their impact on age-related macular degeneration. Prog. Retin. Eye Res..

[195] Ramirez-Pardo I., Villarejo-Zori B., Jimenez-Loygorri J.I., Sierra-Filardi E., Alonso-Gil S., Mariño G., de la Villa P., Fitze P.S., Fuentes J.M., García-Escudero R. (2023). Ambra1 haploinsufficiency in CD1 mice results in metabolic alterations and exacerbates age-associated retinal degeneration. Autophagy.

[196] Lazzeri G., Biagioni F., Fulceri F., Busceti C.L., Scavuzzo M.C., Ippolito C., Salvetti A., Lenzi P., Fornai F. (2018). mTOR Modulates Methamphetamine-Induced Toxicity through Cell Clearing Systems. Oxid. Med. Cell. Longev..

[197] Palikaras K., Lionaki E., Tavernarakis N. (2015). Coordination of mitophagy and mitochondrial biogenesis during ageing in *C. elegans*. Nature.

[198] Zaninello M., Palikaras K., Naon D., Iwata K., Herkenne S., Quintana-Cabrera R., Semenzato M., Grespi F., Ross-Cisneros F.N., Carelli V. (2020). Inhibition of autophagy curtails visual loss in a model of autosomal dominant optic atrophy. Nat. Commun..

[199] Fisher C.R., Shaaeli A.A., Ebeling M.C., Montezuma S.R., Ferrington D.A. (2022). Investigating mitochondrial fission, fusion, and autophagy in retinal pigment epithelium from donors with age-related macular degeneration. Sci. Rep..

[200] Xue C., Yan Z., Cheng W., Zhang D., Zhang R., Duan H., Zhang L., Ma X., Hu J., Kang J. (2025). Curcumin ameliorates aging-induced blood-testis barrier disruption by regulating AMPK/mTOR mediated autophagy. PLoS ONE.

[201] Pendergrass W., Wolf N., Poot M. (2004). Efficacy of MitoTracker Green and CMXrosamine to measure changes in mitochondrial membrane potentials in living cells and tissues. Cytometry.

[202] Ferrucci M., Busceti C.L., Lazzeri G., Biagioni F., Puglisi-Allegra S., Frati A., Lenzi P., Fornai F. (2022). Bacopa Protects against Neurotoxicity Induced by MPP^+^ and Methamphetamine. Molecules.

[203] Marwaha R., Sharma M. (2017). DQ-Red BSA Trafficking Assay in Cultured Cells to Assess Cargo Delivery to Lysosomes. Bio. Protoc..

[204] Isotani S., Hara K., Tokunaga C., Inoue H., Avruch J., Yonezawa K. (1999). Immunopurified mammalian target of rapamycin phosphorylates and activates p70 S6 kinase in vitro. J. Biol. Chem..

[205] McMahon L.P., Choi K.M., Lin T.A., Abraham R.T., Lawrence J.C. (2002). The rapamycin-binding domain governs substrate selectivity by the mammalian target of rapamycin. Mol. Cell. Biol..

[206] Bendayan M., Zollinger M. (1983). Ultrastructural localization of antigenic sites on osmium-fixed tissues applying the protein A-gold technique. J. Histochem. Cytochem..

[207] D’Alessandro D., Mattii L., Moscato S., Bernardini N., Segnani C., Dolfi A., Bianchi F. (2004). Immunohistochemical demonstration of the small GTPase RhoA-A on epoxyresin embedded sections. Micron.

[208] Mattii L., Bianchi F., Falleni A., Frascarelli S., Masini M., Alì G., Chiellini G., Sabbatini A.R.M. (2020). Ultrastructural Localization of Histidine-rich Glycoprotein in Skeletal Muscle Fibers: Colocalization with AMP Deaminase. J. Histochem. Cytochem..

[209] Swanlund J.M., Kregel K.C., Oberley T.D. (2010). Investigating autophagy: Quantitative morphometric analysis using electron microscopy. Autophagy.

[210] Lucocq J.M., Hacker C. (2013). Cutting a fine figure: On the use of thin sections in electron microscopy to quantify autophagy. Autophagy.

[211] Neikirk K., Lopez E.G., Marshall A.G., Alghanem A., Krystofiak E., Kula B., Smith N., Shao J., Katti P., Hinton A. (2023). Call to action to properly utilize electron microscopy to measure organelles to monitor disease. Eur. J. Cell. Biol..

[212] Wagner B., Stirling J., Collinson L., Burrell A. (2025). Ghadially’s Ultrastructural Pathology of the Cell and Matrix.

[213] Ferrucci M., Lenzi P., Lazzeri G., Busceti C.L., Frati A., Puglisi-Allegra S., Fornai F. (2024). Combined light and electron microscopy (CLEM) to quantify methamphetamine-induced alpha-synuclein-related pathology. J. Neural Transm..

[214] Huang C., Wang J.J., Jing G., Li J., Jin C., Yu Q., Falkowski M.W., Zhang S.X. (2015). Erp29 Attenuates Cigarette Smoke Extract-Induced Endoplasmic Reticulum Stress and Mitigates Tight Junction Damage in Retinal Pigment Epithelial Cells. Investig. Ophthalmol. Vis. Sci..

[215] Wagner N., Tsai T., Reinehr S., Theile J., Dick H.B., Joachim S.C. (2024). Retinal debris triggers cytotoxic damage in cocultivated primary porcine RPE cells. Front. Neurosci..

[216] Septinus M., Seiffert W., Zimmermann H.W. (1983). Hydrophobic acridine dyes for fluorescence staining of mitochondria in living cells. 1. Thermodynamic and spectroscopic properties of 10-n-alkylacridine orange chlorides. Histochemistry.

[217] Soultanakis R.P., Melamede R.J., Bespalov I.A., Wallace S.S., Beckman K.B., Ames B.N., Taatjes D.J., Janssen-Heininger Y.M. (2000). Fluorescence detection of 8-oxoguanine in nuclear and mitochondrial DNA of cultured cells using a recombinant Fab and confocal scanning laser microscopy. Free Radic. Biol. Med..

[218] Tiano L., Ballarini P., Santoni G., Wozniak M., Falcioni G. (2000). Morphological and functional changes of mitochondria from density separated trout erythrocytes. Biochim. Biophys. Acta.

[219] Strack A., Duffy C.F., Malvey M., Arriaga E.A. (2001). Individual mitochondrion characterization: A comparison of classical assays to capillary electrophoresis with laser-induced fluorescence detection. Anal. Biochem..

[220] Vizler C., Nagy T., Kusz E., Glavinas H., Duda E. (2002). Flow cytometric cytotoxicity assay for measuring mammalian and avian NK cell activity. Cytometry.

[221] Rodriguez-Enriquez S., Kim I., Currin R.T., Lemasters J.J. (2006). Tracker dyes to probe mitochondrial autophagy (mitophagy) in rat hepatocytes. Autophagy.

[222] Dong H., Cheung S.H., Liang Y., Wang B., Ramalingam R., Wang P., Sun H., Cheng S.H., Lam Y.W. (2013). “Stainomics”: Identification of mitotracker labeled proteins in mammalian cells. Electrophoresis.

[223] Mauro-Lizcano M., Esteban-Martínez L., Seco E., Serrano-Puebla A., Garcia-Ledo L., Figueiredo-Pereira C., Vieira H.L., Boya P. (2015). New method to assess mitophagy flux by flow cytometry. Autophagy.

[224] Marcondes N.A., Terra S.R., Lasta C.S., Hlavac N.R.C., Dalmolin M.L., Lacerda L.A., Faulhaber G.A.M., González F.H.D. (2019). Comparison of JC-1 and MitoTracker probes for mitochondrial viability assessment in stored canine platelet concentrates: A flow cytometry study. Cytometry A.

[225] Chu Y., Park J., Kim E., Lee S. (2021). Fluorescent Materials for Monitoring Mitochondrial Biology. Materials.

[226] Neikirk K., Marshall A.G., Kula B., Smith N., LeBlanc S., Hinton A. (2023). MitoTracker: A useful tool in need of better alternatives. Eur. J. Cell Biol..

[227] Chen C., Li H., Zhang J., Cheng S.C. (2024). Exploring the limitations of mitochondrial dye as a genuine horizontal mitochondrial transfer surrogate. Commun. Biol..

[228] Bergmans J.M.M., van de Westerlo E.M.A., Grefte S., Adjobo-Hermans M.J.W., Koopman W.J.H. (2025). Mitochondrial Morphofunctional Profiling in Primary Human Skin Fibroblasts Using TMRM and Mitotracker Green Co-staining. Methods Mol. Biol..

[229] Giorgi F.S., Saccaro L.F., Busceti C.L., Biagioni F., Fornai F. (2020). Epilepsy and Alzheimer’s Disease: Potential mechanisms for an association. Brain Res. Bull..

[230] Li F., Chen Y., Zheng W., Li K., Chen Z. (2025). Everolimus alleviates cognitive dysfunction in 5×FAD mice by regulating mitochondrial function and oxidative stress. Eur. J. Pharmacol..

[231] Fornai F., Giorgi F.S., Alessandrì M.G., Giusiani M., Corsini G.U. (1999). Effects of pretreatment with N-(2-chloroethyl)-N-ethyl-2-bromobenzylamine (DSP-4) on methamphetamine pharmacokinetics and striatal dopamine losses. J. Neurochem..

[232] Battaglia G., Busceti C.L., Pontarelli F., Biagioni F., Fornai F., Paparelli A., Bruno V., Ruggieri S., Nicoletti F. (2003). Protective role of group-II metabotropic glutamate receptors against nigro-striatal degeneration induced by 1-methyl-4-phenyl-1,2,3,6-tetrahydropyridine in mice. Neuropharmacology.

[233] Schon E., Matheoud D., Przedborski S. (2025). The Mitochondrial Connection in Parkinson’s Disease. Cold Spring Harb. Perspect. Med..

[234] Lazzeri G., Lenzi P., Busceti C.L., Ferrucci M., Falleni A., Bruno V., Paparelli A., Fornai F. (2007). Mechanisms involved in the formation of dopamine-induced intracellular bodies within striatal neurons. J. Neurochem..

[235] Singh D. (2025). Mitochondrial Dysfunction in Neurodegenerative Disorders: Role of Prototype Targeted Drug Delivery Solutions. Curr. Drug Saf..

[236] Biagioni F., Celli R., Giorgi F.S., Nicoletti F., Fornai F. (2022). Perspective on mTOR-dependent Protection in Status Epilepticus. Curr. Neuropharmacol..

[237] Giorgi F.S., Biagioni F., Lenzi P., Frati A., Fornai F. (2015). The role of autophagy in epileptogenesis and in epilepsy-induced neuronal alterations. J. Neural. Transm..

[238] Ryskalin L., Gaglione A., Limanaqi F., Biagioni F., Familiari P., Frati A., Esposito V., Fornai F. (2019). The Autophagy Status of Cancer Stem Cells in Gliobastoma Multiforme: From Cancer Promotion to Therapeutic Strategies. Int. J. Mol. Sci..

[239] Fulceri F., Biagioni F., Ferrucci M., Lazzeri G., Bartalucci A., Galli V., Ruggieri S., Paparelli A., Fornai F. (2007). Abnormal involuntary movements (AIMs) following pulsatile dopaminergic stimulation: Severe deterioration and morphological correlates following the loss of locus coeruleus neurons. Brain Res..

[240] Squitieri F., Falleni A., Cannella M., Orobello S., Fulceri F., Lenzi P., Fornai F. (2010). Abnormal morphology of peripheral cell tissues from patients with Huntington disease. J. Neural. Transm..

[241] Limanaqi F., Biagioni F., Mastroiacovo F., Polzella M., Lazzeri G., Fornai F. (2020). Merging the Multi-Target Effects of Phytochemicals in Neurodegeneration: From Oxidative Stress to Protein Aggregation and Inflammation. Antioxidants.

[242] Limanaqi F., Biagioni F., Gaglione A., Busceti C.L., Fornai F. (2019). A Sentinel in the Crosstalk Between the Nervous and Immune System: The (Immuno)-Proteasome. Front. Immunol..

