# Supplementary material for: Modulation of mTOR Within Retinal Pigment Epithelium Affects Cell Viability and Mitochondrial Pathology
Source: Int J Mol Sci. 2025 Sep 26;26(19):9442. doi: 10.3390/ijms26199442 (PMC12524646; doi:10.3390/ijms26199442)
Supplement: Supplementary file 1 [file ijms-26-09442-s001.zip › ijms-3822096-supplementary.pdf]

Supplementary Table S1. Comparison between Tomm20-immunopositive and MTR-G-positive areas*

**Experimental groups**	**Area of Tomm20 Immunofluorescence (m^2)**	**Area of MTR-G Immunofluorescence (m^2)**	***P* values**
Control	300.5±7.6	316.1±11.0	0.9999
3-MA	90.3±3.2	119.3±4.9	0.9626
Curcumin	330.3±10.0	347.1±12.1	0.9997
Curcumin+3-MA	240.4±10.0	289.9±14.0	0.3441
Rapamycin	298.5±10.3	323.0±12.8	0.9903
Rapamycin+3-MA	215.0±8.1	252.4±10.3	0.7974

*Values were compared by using one-way analysis of variance, ANOVA, followed by Scheffè's post hoc analysis. Differences between the groups were considered to be significant when the null hypothesis (H_0) was $P < 0.05$.
